# Reduced Stiffness Modeling of Perforated Thin-Walled C- and Z-Sections with Warping Effects

**DOI:** 10.3390/ma19204372

**Published:** 2026-10-17

**Authors:** Jolanta Pozorska, Zbigniew Pozorski, Tomasz Garbowski

**Affiliations:** 1 Institute of Mathematics, Poznan University of Technology, 61-131 Poznan, Poland; jolanta.pozorska@put.poznan.pl; 2 Institute of Structural Analysis, Poznan University of Technology, 61-131 Poznan, Poland; zbigniew.pozorski@put.poznan.pl; 3 Department of Biosystems Engineering, Poznan University of Life Sciences, 60-627 Poznan, Poland

**Keywords:** cold-formed steel, thin-walled sections, web openings, static condensation, reduced-order modeling, warping, generalized beam element, stiffness matrix, proper orthogonal decomposition, surrogate modeling

## Abstract

Web openings in cold-formed steel members modify not only individual stiffness components but also the interactions between bending, shear, torsion, and warping. Capturing these effects in a compact structural model is essential for repeated assessment of deflections and twist as opening geometry changes. This study develops a reduced stiffness formulation for perforated C- and Z-sections by combining shell finite element analysis, static condensation, and interface kinematics enriched with sectorial warping. The resulting 14 × 14 element matrix transfers the local influence of an opening into a beam-type representation suitable for assembling members with repeated or non-uniform perforations. Geometry-dependent stiffness variations are approximated using proper orthogonal decomposition and Gaussian process regression in an energy-scaled logarithmic matrix space, preserving rigid-body modes and positive deformational stiffness. Verification combines comparisons against continuous shell models with independent analytical torsion benchmarks for unperforated sections. For the investigated members, direct reduction decreases the number of degrees of freedom by factors of 662–794, with displacement and twist profile errors below 1.33%. A surrogate using three coefficients per section family reproduces the tested member responses for opening geometries excluded from training with errors below 0.47%. The framework provides a compact basis for preliminary stiffness assessment and opening-geometry studies within the small-displacement, pre-buckling elastic regime.

## 1. Introduction

Cold-formed thin-walled steel members offer a favorable strength-to-weight ratio, manufacturing flexibility, and high structural efficiency, which has led to their widespread use in building systems, storage structures, lightweight frames, and other engineering applications. In many practical configurations, C- and Z-sections contain web openings introduced for installation purposes, connections, weight reduction, thermal considerations, or technological requirements. Such perforations interrupt the otherwise prismatic geometry and may considerably alter the mechanical response of the member. Early investigations therefore focused primarily on the degradation of strength and stability caused by openings. Miller and Peköz [1] examined local buckling of perforated wall studs, while Shan et al. [2] and LaBoube et al. [3] investigated the interaction of bending, shear, and web openings in cold-formed C-sections. Davies et al. [4] subsequently addressed perforated sections subjected to combined axial loading and bending, highlighting the additional complexity introduced by regularly distributed holes.

A substantial part of the subsequent research has remained focused on strength-oriented representations of perforated members. The concept of an equivalent reduced web thickness was proposed as a practical means of representing regularly perforated channel sections [5], whereas detailed studies examined elastic buckling of plates with holes [6] and incorporated the influence of openings into the Direct Strength Method for cold-formed steel columns [7]. Web crippling and its interaction with perforations were investigated experimentally and numerically by Uzzaman et al. [8,9]. Alternative numerical approaches have also been developed for stability assessment, including finite-strip formulations for perforated rack columns [10] and thin-walled columns with repeated perforation patterns [11]. These investigations established that the influence of an opening cannot generally be represented solely by the loss of cross-sectional area, since its geometry and location affect local, distortional, and global deformation mechanisms.

Strength-oriented methods, including the Direct Strength Method and effective-width approaches, address resistance and stability but do not directly supply the full coupled elastic operator of an arbitrary perforated segment. A separate stiffness description is useful when the engineering question concerns deflection, twist, or redistribution of elastic actions. Examples include service openings in wall studs, eccentric loading of roof purlins, and stiffness screening of perforated rack or photovoltaic-support members. The present model is intended to support such elastic analyses; it is not a replacement for their strength checks.

Further work has therefore increasingly addressed interactions between perforation geometry and specific structural modes. Cai and Moen [12] employed Generalized Beam Theory (GBT) to investigate elastic buckling of members containing rectangular holes, while Wang and Young [13] studied the flexural behavior of built-up cold-formed sections with web perforations. Distortional buckling of channel beams with circular openings was analyzed by Yuan et al. [14], and the effects of edge-stiffened openings on compressed channel sections were investigated by Chen et al. [15]. Pham et al. [16] focused on shear strength and design of channels with web holes, whereas Yu et al. [17] examined distortional buckling under distributed transverse loading. More recent studies have continued this trend toward increasingly detailed treatment of perforation-induced instability and strength reduction, including analytical formulations for distortional buckling [18], combined compression and bending [19], and web-perforated lipped channels [20]. The broad scope of current research in this field has recently been summarized by Zeng et al. [21], confirming that strength, buckling, and ultimate resistance remain the dominant topics in studies of perforated cold-formed members.

An important parallel development concerns reduced one-dimensional representations capable of reproducing complex thin-walled behavior at substantially lower computational cost than detailed shell models. GBT provides an especially powerful framework because global, local, distortional, and warping deformation modes can be introduced explicitly into a beam-type description [22,23]. Its extension to perforated members has led to several sophisticated formulations. Bonada et al. [24] incorporated perforation effects into geometrically nonlinear GBT analysis of rack columns, while Duan and Zhao [25] developed an extended first-order GBT formulation in which enrichment functions describe displacement discontinuities associated with arbitrary holes. This framework was subsequently extended to linear buckling [26], elastoplastic analysis [27], edge-stiffened openings [28], and, most recently, large-deformation analysis [29]. Dynamic behavior of perforated rack members has also been successfully treated within the GBT framework [30]. These approaches demonstrate that advanced beam theories can retain much of the mechanical richness of shell models while significantly reducing the number of degrees of freedom.

C-sections are used, for example, as studs and channel members, while Z-sections are common in purlin systems. For the geometrically balanced sections studied here, mirror symmetry of the C-section and point symmetry of the Z-section produce different coupling and warping fields even when gross dimensions are identical. Comparing both therefore tests whether a common reduction strategy can represent distinct open-section kinematics, rather than only two values of the same scalar stiffness.

A different reduction philosophy consists of deriving an equivalent one-dimensional description directly from a detailed numerical model rather than prescribing a specialized analytical representation of the perforation. Static condensation, originating from classical matrix reduction concepts [31], provides a natural basis for such an approach because internal degrees of freedom can be eliminated while preserving the force–displacement relationship at selected interfaces. Shell-to-beam numerical homogenization has previously been used to determine equivalent stiffnesses of thin-walled perforated beams [32] and subsequently combined with parametric optimization and soft-computing techniques [33]. Related studies demonstrated that the same energy-equivalence philosophy can accommodate local geometric imperfections [34], perforation and creasing in layered structures [35], and non-classical shear effects that are difficult to estimate using elementary sectional formulas [36]. These results suggest that detailed local mechanics can be transferred effectively to computationally inexpensive structural representations without explicitly deriving separate analytical correction factors for each deformation mechanism.

Other recent approaches also point toward homogenized or equivalent descriptions of perforated members. Dong et al. [37], for example, proposed a homogenized model accounting for the enhanced shear deformation of perforated steel beams and demonstrated that opening geometry can be incorporated through equivalent stiffness measures. However, equivalent sectional properties such as *EA*, *EI*, *GA*, or *GJ* characterize individual deformation mechanisms separately and may not retain the complete coupling structure of an arbitrary perforated thin-walled segment. This limitation becomes particularly relevant for open C- and Z-sections, where shear, bending, torsion, and cross-sectional warping may interact and where the presence of a web opening may modify not only individual stiffness terms but also their mutual couplings.

The present study therefore adopts a different perspective. Rather than deriving isolated effective sectional properties or introducing the perforation directly into a predefined beam theory, a detailed shell segment containing an opening is first reduced by static condensation to its end interfaces. The condensed response is then projected onto a beam-type kinematic basis enriched by a sectorial warping mode, resulting in a compact element stiffness matrix *K_e_*. Consequently, the local shell deformation around the opening is retained energetically in a generalized one-dimensional element that can subsequently be assembled in the same manner as conventional finite elements. The perforation effect is expressed through an opening-induced stiffness correction, $\Delta K_{e}=K_{e}-K_{e}^{0}$, with respect to the corresponding unperforated segment.

A further objective is to determine whether this matrix correction possesses an intrinsically low-dimensional structure. Proper orthogonal decomposition (POD) has proven effective in reducing parametrically varying structural systems [38] and in constructing reduced representations of modular structures whose independently generated substructures are subsequently assembled [39]. Here, POD is applied not to displacement snapshots but to dimensionless stiffness-correction matrices. If only a limited number of dominant matrix modes is required, the geometry-dependent reduced stiffness can be reconstructed from a small set of modal coefficients, which may subsequently be predicted using Gaussian process regression [40]. The resulting framework establishes a direct path from detailed shell mechanics to a warping-enriched beam model whose element stiffness can be rapidly adapted to different opening geometries and assembled into long perforated C- and Z-section members. This provides the basis for computationally efficient structural analysis while preserving the essential stiffness modifications and coupling effects induced by web perforations. Warping-dominated torsion in cold-formed lipped channels [41] motivates the retained sectorial coordinate.

The study has three application targets: (i) preliminary elastic displacement and twist assessment of members containing repeated or non-uniform openings; (ii) rapid screening and sensitivity analysis of opening dimensions within a verified geometric family; and (iii) reusable stiffness operators for repeated analyses in optimization or uncertainty-propagation workflows. The first target is assessed by the member examples and the second by the matrix database and surrogate. The third is an intended use enabled by the reduced representation, not an optimization or probabilistic application demonstrated in this paper. Centered rectangular openings are treated as a methodological proof of concept.

## 2. Materials and Methods

The proposed formulation replaces a detailed shell representation of a perforated thin-walled segment by a compact one-dimensional finite element whose stiffness matrix retains the energetic influence of the opening, including bending, shear, torsion, cross-sectional warping, and the couplings between these deformation mechanisms. The method is formulated for cold-formed C- and Z-sections, although the reduction procedure itself is not restricted to these cross-sectional families. A local Cartesian system *x*-*y*-*z* is adopted, with *x* aligned with the member axis and *y* and *z* lying in the cross-sectional plane. The reference point *O* of each intact end section is located at the centroid of the corresponding unperforated cross-section. The same reference system is used for the perforated and unperforated segments so that the resulting stiffness matrices can be compared componentwise.

**Figure 1:**
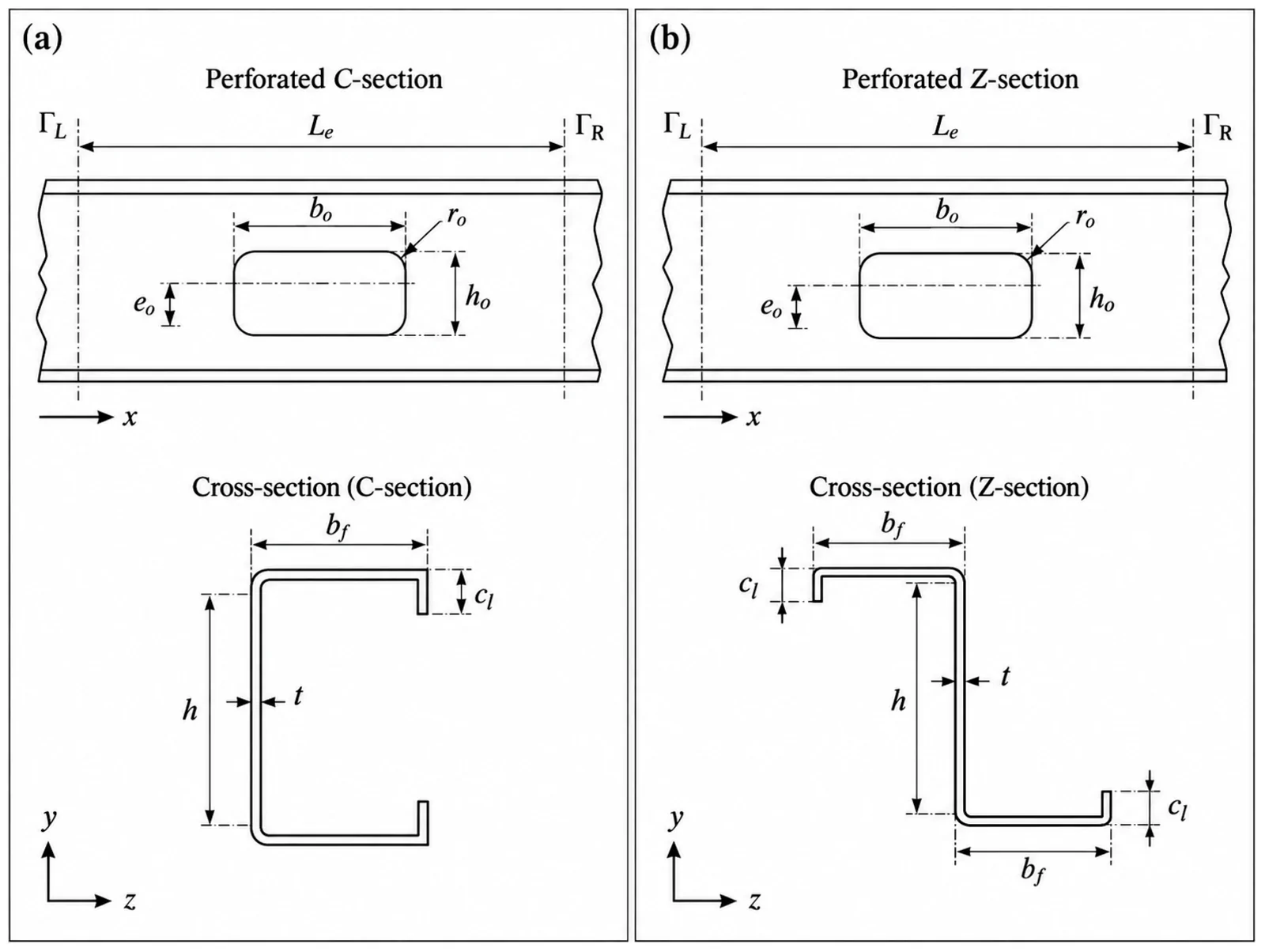
Geometry of the perforated: (**a**) C- and (**b**) Z-section segments; local coordinate system, intact end interfaces *Γ_L_* and *Γ_R_*, and parameters describing the web opening.

A computational segment of length *L_e_* contains one web opening and is bounded by two intact cross-sections, denoted by *Γ_L_* and *Γ_R_*. For regularly perforated members, *L_e_* is equal to the opening pitch *p*, and the opening is placed at the center of the segment. For non-uniform perforation patterns, the interfaces may be positioned at any intact cross-sections between adjacent openings, provided that the same interface definition is used in the detailed and reduced models. Figure 1 illustrates the adopted segment, coordinate system, opening parameters, and end interfaces. The geometric notation used throughout the formulation is summarized in Table 1.

Unless stated otherwise, the numerical analyses were performed for lipped C- and Z-sections with *h* = 150 mm, *b_f_* = 60 mm, *c_l_* = 20 mm, and *t* = 2 mm. The reduced-element length was *L_e_* = 240 mm. The material was assumed to be linear elastic with *E* = 210 GPa and *ν* = 0.30. The same nominal dimensions were adopted for the C- and Z-section families so that differences in the reduced response resulted from cross-sectional arrangement rather than from changes in gross dimensions.

The geometry of an opening is represented by a dimensionless parameter vector. For a fixed cross-sectional family, a convenient generic description is

**ξ** = [*α*,*β*,*ε*,*ρ*,*λ*]*^T^*, (1)

The parameter vector in Equation (1) describes possible extensions of the framework. The present numerical database, however, contains only centered rectangular openings in the two fixed section families. This choice isolates the effects of opening height and length without simultaneously varying eccentricity, edge stiffening, or opening topology. For another opening family, a conforming shell mesh and a suitable continuous parameterization would first be required. The dimensionless corrections would then be recomputed, the POD reconstruction errors reassessed, and the regression models retrained. Separate mappings are appropriate for distinct section or opening families; their categorical labels should not be interpreted as numerical distances. Neither the present POD rank nor its reported accuracy is assumed to transfer to other opening families.

The material is homogeneous, isotropic, and linearly elastic. Yielding, residual stresses, geometric imperfections, and material nonlinearity are excluded because the target is initial elastic stiffness. The shell discretization uses six DOFs per node and conforming rectangular panels. Opening edges are inserted into the longitudinal and web grids. Mesh convergence is evaluated using both the complete dimensionless reduced matrix and individually specified energy states; a small matrix-norm difference alone is not taken as convergence of every component.

The numerical reference is a continuous shell model of the complete member, including its web, flanges, lips, and explicit openings. Flat rectangular four-node Reissner–Mindlin elements use 2 × 2 membrane and bending integration, MITC4-type assumed transverse shear on these rectangular panels [42], a shear factor of 5/6, and a consistent drilling penalty of 10^−4^*Gt*. Opening edges, fold lines, and extraction cuts are included exactly in the mesh. Interior cut sections share the same shell translations and rotations and are not diaphragms. The actual C- and Z-section meshes follow this discretization. A common cross-sectional node grid is used for all segments of each non-uniform member, preventing incompatible meshes at internal cuts.

For a given detailed segment, the linear finite element equilibrium equations are written as

**Ku** = **f**, (2)

where **K** is the assembled shell stiffness matrix, **u** is the vector of nodal degrees of freedom, and **f** is the corresponding nodal force vector. The degrees of freedom are partitioned into retained interface translations, denoted by **u***_Γ_*, and eliminated degrees of freedom **u***_i_*. Importantly, only the three translational degrees of freedom of the nodes located on *Γ_L_* ∪ *Γ_R_* are retained. All remaining degrees of freedom, including the rotational shell degrees of freedom at the interfaces, are included in **u***_i_*. This choice avoids prescribing an artificial shell-director rotation at the reduced interfaces and allows the local shell rotations to relax consistently with the imposed translational interface kinematics. The partitioned system is therefore

(3) $$\left[\begin{array}{cc} K_{\Gamma \Gamma } & K_{\mathrm{\Gamma i}}\\ K_{\mathrm{i\Gamma}} & K_{\mathrm{ii}} \end{array}\right]\left[\begin{array}{c} u_{\Gamma }\\ u_{i} \end{array}\right]=\left[\begin{array}{c} f_{\Gamma }\\ 0 \end{array}\right],$$

where no external forces are applied to the eliminated internal degrees of freedom during the reduction. Static condensation [31] gives the exact interface stiffness associated with the retained translations,

(4) $$K_{\Gamma }=K_{\Gamma \Gamma }-K_{\mathrm{\Gamma i}}K_{\mathrm{ii}}^{-1}K_{\mathrm{i\Gamma}},$$

so that **f***_Γ_* = **K***_Γ_***u***_Γ_*. In the numerical implementation, the inverse in Equation (4) is not formed explicitly; the required products are obtained by solving linear systems with **K***_ii_*. The condensation preserves the exact static force-displacement relation at the retained interface translations. At this stage, no beam assumption has yet been introduced and **K***_Γ_* still allows arbitrary translational deformation patterns of both end cross-sections.

The second reduction step introduces six rigid-section modes and one axial sectorial warping mode for the intact section. The sectorial function is evaluated by exact line integration on the piecewise-straight contour, with three-point Gauss integration of the orthogonalization products. Its sign is fixed by a positive value at the first contour endpoint. It is then interpolated at interface nodes, so refinement and changes in the opening geometry do not redefine the physical basis. Local deformation is allowed inside the shell segment; only the retained interface trace is restricted. An intact interface alone does not establish the adequacy of this restriction. The near-interface study quantifies both the global response error and the omitted interface deformation.

The preliminary sectorial coordinate *ω*_0_(*s*), defined along the mid-line coordinate *s* of the thin-walled cross-section, is evaluated from

(5) $$\omega_{0}\left(s\right)=\int_{s_{0}}^{s}\left[y\left(\hat{s}\right)\frac{\mathrm{dz}}{d\hat{s}}-z\left(\hat{s}\right)\frac{\mathrm{dy}}{d\hat{s}}\right]d\hat{s},$$

with *y* and *z* measured from the adopted cross-sectional reference point. Since the sectorial coordinate is defined up to additive rigid and linear components, it is orthogonalized with respect to the axial extension and the two bending fields. The corrected warping function is written as

*ω*(*s*) = *ω*_0_(*s*) − *c*_0_ − *c_y_y*(*s*) − *c_z_z*(*s*), (6)

where *c*_0_, *c_y_*, and *c_z_* are selected such that

(7) $$\int_{A}\omega \mathrm{dA}=0,\;\;\int_{A}\mathrm{y\omega}\;\mathrm{dA}=0,\;\;\int_{A}\mathrm{z\omega}\;\mathrm{dA}=0,$$

**Figure 2:**
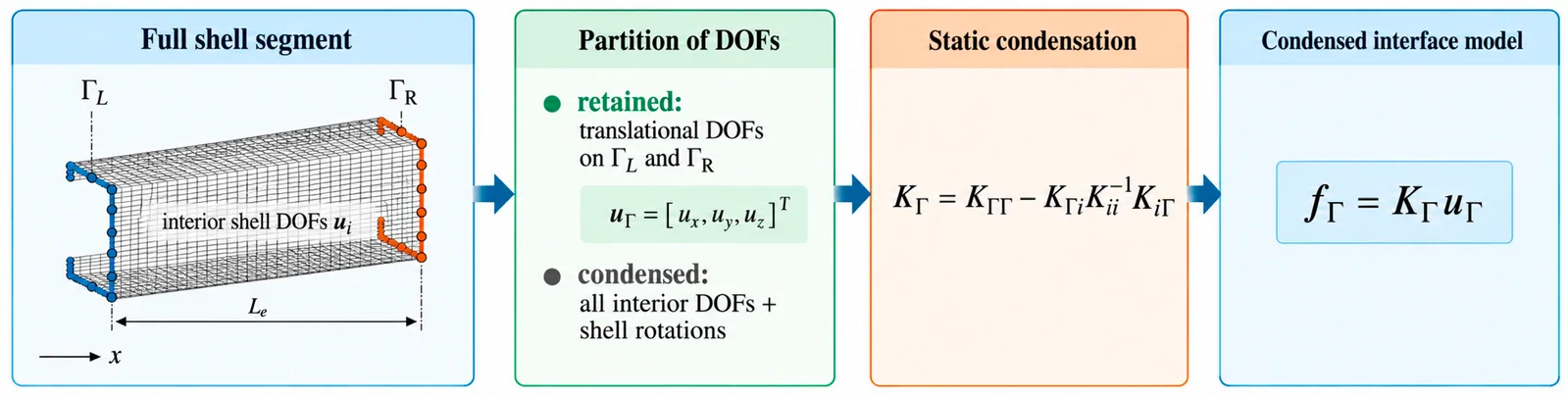
Static-condensation stage of the shell-to-reduced-element formulation: full shell segment, partition into retained interface translations and eliminated degrees of freedom, static condensation, and the condensed interface relation ***f****_Γ_* = ***K****_Γ_**u**_Γ_*.

The three coefficients can be obtained directly from the small linear system

(8) $$\int_{A}\left[\begin{array}{ccc} 1 & y & z\\ y & y^{2} & \mathrm{yz}\\ z & \mathrm{yz} & z^{2} \end{array}\right]\mathrm{dA}\left[\begin{array}{c} c_{0}\\ c_{y}\\ c_{z} \end{array}\right]=\int_{A}\left[\begin{array}{c} 1\\ y\\ z \end{array}\right]\omega_{0}\;\mathrm{dA},$$

where the area integrals are evaluated over the thin-walled cross-section, equivalently as thickness-weighted line integrals along its mid-line. Orthogonalization in Equations (6)–(8) prevents the warping coordinate from duplicating the constant axial or linear bending modes. A consistent sign convention is enforced for all models by requiring the value of *ω* at a selected reference flange tip to be positive. Figure 2 summarizes the static-condensation stage preceding the projection onto the generalized interface basis.

The three conditions in Equation (7) are orthogonality to the axial fields 1, *y*, and *z*, respectively; the second coordinate is the Latin *y*. They remove extension and the two linear bending components, irrespective of whether the selected axes are principal axes. Let **v** = (1,*y*,*z*)*^T^* and $M=\int_{A}vv^{T}\;\mathrm{dA}$. For any nonzero coefficient vector **c**, $c^{T}\mathrm{Mc}=\int_{A}{\left(c_{0}+c_{y}y+c_{z}z\right)}^{2}\;\mathrm{dA}>0$ provided that the cross-sectional support is not contained in one straight line and the thickness weights are positive. Thus the C- and Z-section Gram matrices are nonsingular without requiring a symmetry axis or a special pole. Centering sets the first moments to zero but is not the reason for nonsingularity. Principal axes may diagonalize the bending block; they are not required for the projection. Very poorly conditioned discrete geometries can instead be treated using a weighted QR or SVD projection.

At each end of the segment, the generalized displacement vector is defined as

(9) $$q={\left[u,v,w,\theta_{x},\theta_{y},\theta_{z},\gamma_{\omega }\right]}^{T},$$

where *u*, *v*, and *w* are translations of the reference point, *θ_x_*, *θ_y_*, and *θ_z_* are cross-sectional rotations, and *γ_ω_* is the amplitude of the sectorial warping mode. Since [*ω*] = *L*^2^, the warping amplitude has dimension [*γ_ω_*] = *L*^−1^, ensuring that *ωγ_ω_* has the dimension of displacement. The formulation does not impose the classical Vlasov relation between *γ_ω_* and the spatial derivative of twist inside the reduced segment; instead, *γ_ω_* is treated as an independent interface coordinate. The relation between torsional rotation, warping, and the corresponding generalized forces is therefore inherited directly from the condensed shell stiffness.

Equation (10) assumes infinitesimal rigid-section motion in the transverse plane and axial sectorial warping. The additional mode therefore enters only the axial displacement. Transverse distortion and wall-normal displacement are not independent interface coordinates, although they remain available inside the shell segment. The weighted residual $∥u_{\Gamma }^{\mathrm{FE}}-\Phi q^{\mathrm{FE}}{∥}_{W}/∥u_{\Gamma }^{\mathrm{FE}}{∥}_{W}$ quantifies the part of the computed interface translation field omitted by this basis. It is evaluated without constraining interior sections of the reference shell. This residual and the twist error are evaluated as the intact ligament beside a large opening is shortened.

For an interface node *j* located at (*y_j_*,*z_j_*), the translational displacement is expressed as

(10) $$\left[\begin{array}{c} u_{x,j}\\ u_{y,j}\\ u_{z,j} \end{array}\right]=B_{j}q,\;\;B_{j}=\left[\begin{array}{ccccccc} 1 & 0 & 0 & 0 & z_{j} & -y_{j} & \omega_{j}\\ 0 & 1 & 0 & -z_{j} & 0 & 0 & 0\\ 0 & 0 & 1 & y_{j} & 0 & 0 & 0 \end{array}\right],$$

which combines rigid translation, rigid cross-sectional rotation, and axial sectorial warping. The matrices **B***_j_* are assembled for all translational degrees of freedom at a given interface to obtain **Φ***_L_* and **Φ***_R_*. For the complete two-ended segment,

(11) $$u_{\Gamma }=\Phi_{e}q_{e},\;\;\Phi_{e}=\left[\begin{array}{cc} \Phi_{L} & 0\\ 0 & \Phi_{R} \end{array}\right],\;\;q_{e}=\left[\begin{array}{c} q_{L}\\ q_{R} \end{array}\right],$$

where **q***_e_* ∈ ℝ^14^. The strain energy stored in the statically condensed shell segment is

(12) $$U_{e}=\frac{1}{2}u_{\Gamma }^{T}K_{\Gamma }u_{\Gamma }=\frac{1}{2}q_{e}^{T}K_{e}q_{e},$$

and energy equivalence immediately yields the reduced element stiffness matrix

(13) $$K_{e}=\Phi_{e}^{T}K_{\Gamma }\Phi_{e},$$

with **K***_e_* ∈ ℝ^14×14^. Equation (13) is the central shell-to-beam reduction used in this study. For comparison, a classical six-degree-of-freedom interface model is obtained simply by deleting the warping column from **Φ***_L_* and **Φ***_R_*, leading to a 12 × 12 element matrix. Table 2 summarizes both reduced kinematic bases. Their comparison provides a direct quantitative measure of the improvement associated with the single additional warping coordinate.

The effect of constraining retained rotations follows from minimization. Write the locally condensed energy as *U*(**t**,**r**), where **t** denotes interface translations and **r** the remaining shell rotations. For **t** = **Φ***_t_***q**, relaxation gives *U*_rel_(**q**) = min**_r_***U*(**Φ***_t_***q**,**r**). A prescribed rotational map instead gives *U*_tie_(**q**) = *U*(**Φ***_t_***q**,**Ψq**) ≥ *U*_rel_(**q**). A scalar illustration is *U*(*a*,*b*) = 1/2*ka*^2^ + 1/2*c*(*b* − *da*)^2^, with *k*,*c* > 0: minimizing over *b* gives stiffness *k*, whereas imposing *b* = 0 gives *k* + *cd*^2^. This explains how a restrictive projection can stiffen a model despite retaining more shell variables before projection. It does not establish which interface treatment best reproduces a continuous shell assembly.

Although Equations (2)–(13) are written in terms of explicit shell stiffness matrices, direct access to the complete assembled matrix is not required. An equivalent implementation can identify **K***_e_* through a sequence of unit generalized-displacement analyses. If **e***_j_* denotes the *j*-th unit vector in the 14-dimensional generalized coordinate space, the boundary translations **u***_Γ_* = **Φ***_e_***e***_j_* are prescribed, the shell problem is solved with all internal degrees of freedom free to equilibrate, and the resulting interface reactions $f_{\Gamma }^{\left(j\right)}$ are projected back to generalized forces according to

(14) $$f_{e}^{\left(j\right)}=\Phi_{e}^{T}f_{\Gamma }^{\left(j\right)},$$

The reduced stiffness matrix is then assembled columnwise as

(15) $$K_{e}=\left[f_{e}^{\left(1\right)},f_{e}^{\left(2\right)},\ldots ,f_{e}^{\left(14\right)}\right],$$

**Figure 3:**
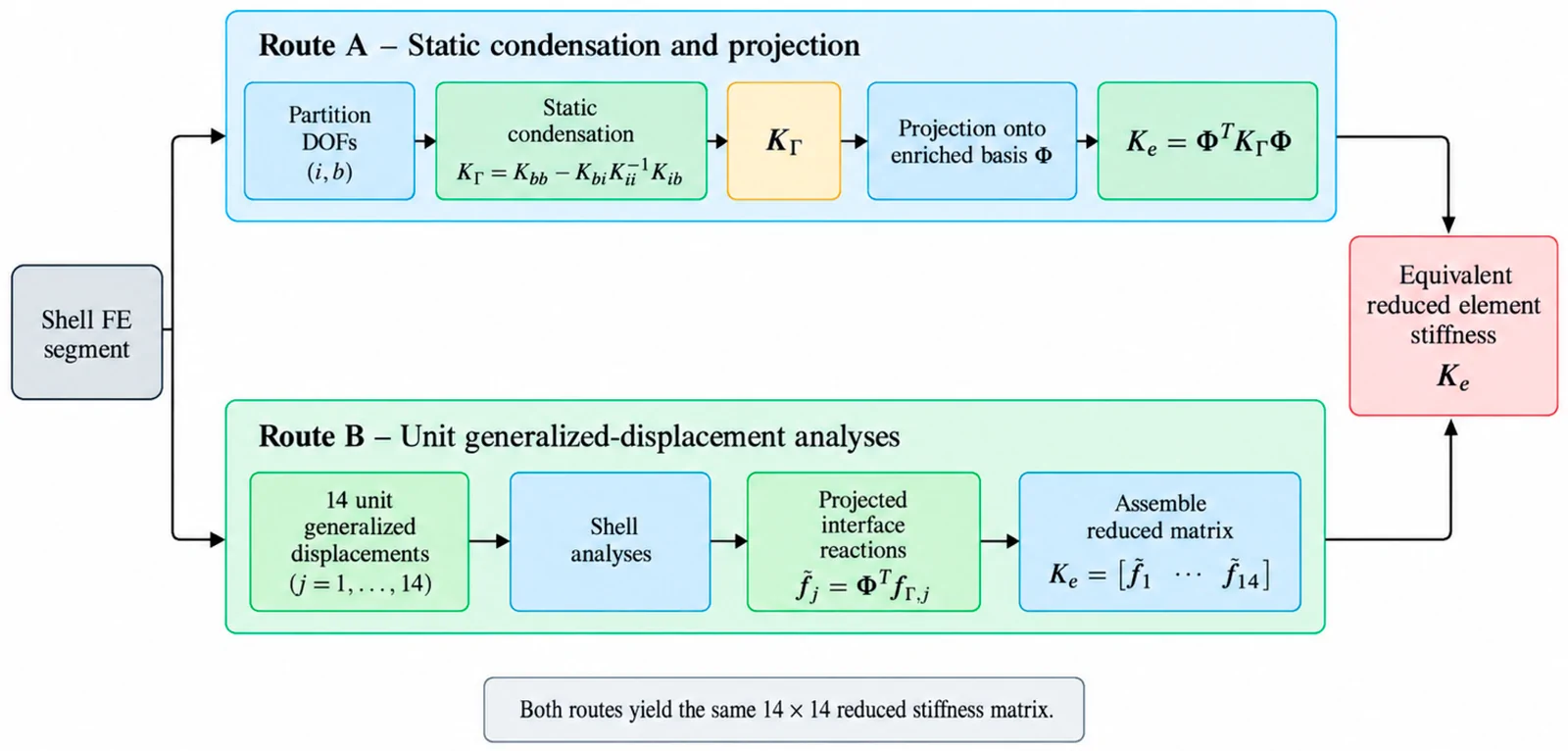
Shell-to-reduced-element workflow. Route A uses explicit static condensation to obtain **K***_Γ_* followed by projection onto the enriched interface basis. Route B identifies the same **K***_e_* from fourteen unit generalized-displacement analyses and projected interface reactions.

The two routes shown in Figure 3 are algebraically equivalent for identical meshes, bases, and boundary enforcement. In Route B, each unit generalized displacement is imposed, the remaining shell degrees of freedom are solved, and the reactions are projected onto the same interface basis. Four same-mesh comparisons, covering solid and perforated C- and Z-sections, gave dimensionless relative matrix differences no larger than 1.08 × 10^−13^. Independently evaluating the equilibrated shell energy gave differences no larger than 8.34 × 10^−14^. These checks verify the condensation/reaction implementation, rather than the underlying shell theory.

For every perforated segment, a geometrically identical reference segment without an opening is reduced using the same interfaces, mesh convention, reference system, and warping basis. Its stiffness matrix is denoted by $K_{e}^{0}$. The mechanical influence of the opening is represented by the signed stiffness-correction operator

(16) $$\Delta K_{e}\left(\xi \right)=K_{e}\left(\xi \right)-K_{e}^{0},$$

so that $K_{e}\left(\xi \right)=K_{e}^{0}+\Delta K_{e}\left(\xi \right)$. Using the correction rather than the total matrix separates the known stiffness of the prismatic unperforated segment from the geometry-dependent perturbation produced by the opening and substantially improves the numerical conditioning of the subsequent reduced-order representation.

Because the generalized coordinates have different physical dimensions, POD must not be applied directly to the raw entries of **K***_e_*. A dimensionless energetic scaling is therefore introduced. With the characteristic length *L*_∗_ = *h*, the scaling matrix for one end is

(17) $$S_{7}=\mathrm{diag}\left(L_{*},L_{*},L_{*},1,1,1,L_{*}^{-1}\right),\;\;S_{e}=\mathrm{diag}\left(S_{7},S_{7}\right),$$

such that $q_{e}=S_{e}{\overset{‾}{q}}_{e}$, where ${\overset{‾}{q}}_{e}$ is dimensionless. Using *EA*_0_*L*_∗_ as the reference energy scale, where *A*_0_ is the gross area of the unperforated cross-section, the dimensionless stiffness matrix is defined as

(18) $${\overset{‾}{K}}_{e}=\frac{S_{e}^{T}K_{e}S_{e}}{EA_{0}L_{*}},\;\;\Delta {\overset{‾}{K}}_{e}={\overset{‾}{K}}_{e}-{\overset{‾}{K}}_{e}^{0},$$

The normalization follows directly from strain-energy equivalence. Substitution of $q_{e}=S_{e}{\overset{‾}{q}}_{e}$ into Equation (12) gives $U_{e}/\left(EA_{0}h\right)=1/2{\overset{‾}{q}}_{e}^{T}{\overset{‾}{K}}_{e}{\overset{‾}{q}}_{e}$, and solving Equation (18) for the dimensional matrix gives Equation (29). Here $A_{0}=\int t\left(s\right)\;\mathrm{ds}$ is the gross area of the intact reference section, used for both solid and perforated segments. It is not the remaining area through the hole. The reference length is *h*, not the element length *L_e_*; variation of *L_e_* remains represented through *λ* = *L_e_*/*h*. Translational, rotational, and warping coordinates are scaled by *h*, 1, and 1/*h*, respectively. Their conjugate forces have dimensions force, force times length, and force times length squared. Consequently, the translational, rotational, and warping diagonal blocks of the dimensional matrix have units force/length, force times length, and force times length cubed. A dimensionless matrix entry is the generalized force generated by a unit dimensionless coordinate while the others are held fixed. Scaling makes the matrix norm independent of the chosen unit system, but does not make it a unique physical error measure for all load cases. All matrix errors and POD snapshots use this same scaling and reference section.

POD is evaluated separately for C and Z and for the combined correction database. The combined basis is used for the linear-surrogate baseline, while the final logarithmic predictor uses separate reference-energy-scaled bases. The symmetric vectorization maps each correction to its upper-triangular entries, multiplying off-diagonal entries by $\sqrt{2}$ so that its Euclidean norm equals the matrix Frobenius norm. Thus,

(19) $$d_{i}=\mathrm{svec}\left(\Delta {\overset{‾}{K}}_{e}^{\left(i\right)}\right)\in R^{105},\;\;{∥d_{i}∥}_{2}={∥\Delta {\overset{‾}{K}}_{e}^{\left(i\right)}∥}_{F},$$

for the proposed 14 × 14 element. For *N* sampled geometries, the stiffness-correction vectors are collected directly in the snapshot matrix

*D* = [*d*_1_, *d*_2_, …, *d_N_*]. (20)

No mean subtraction is applied because the zero correction has a direct mechanical meaning and corresponds to the unperforated reference segment. The singular value decomposition is therefore performed as

*D* = *UΣV^T^*. (21)

The number *R* of retained modes is selected from

(22) $$\eta_{R}=\frac{\sum_{r=1}^{R}\sigma_{r}^{2}}{\sum_{r=1}^{R_{\max}}\sigma_{r}^{2}}\geq 0.999.$$

The POD coordinates associated with sample *i* are

(23) $$a_{i}=U_{R}^{T}d_{i},$$

and the corresponding rank-*R* reconstruction is

(24) $$d_{i}^{\left(R\right)}=U_{R}a_{i}=\sum_{r=1}^{R}a_{\mathrm{ir}}u_{r}.$$

**Figure 4:**
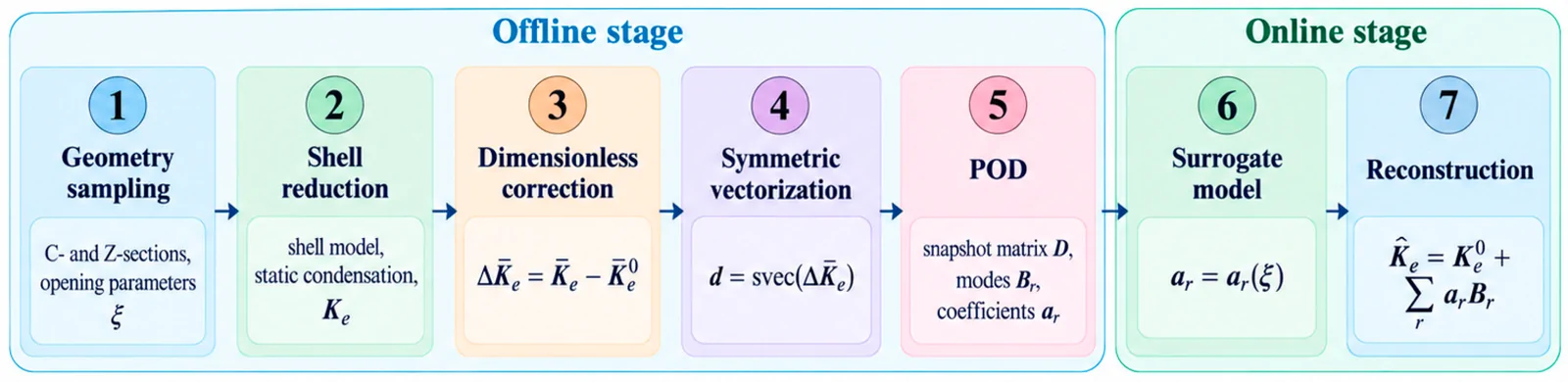
Low-dimensional representation of the opening-induced stiffness correction: geometry sampling, shell reduction, dimensionless matrix correction, symmetric vectorization, POD, surrogate prediction of modal coefficients, and reconstruction of ${\hat{K}}_{e}$. The linear reconstruction is the diagnostic baseline; the final predictor uses the energy-scaled logarithmic mapping in Equations (29a) and (29b).

Here, *U_R_* contains the first *R* POD stiffness-correction modes. For matrix representation, the *r*-th POD stiffness-correction mode is defined as **M***_r_* = svec^−1^(**u***_r_*), so that $\Delta {\bar{K}}_{e}^{\left(R\right)}=\sum_{r=1}^{R}a_{r}M_{r}$. Figure 4 summarizes the complete reduction and reconstruction workflow.

Separate scalar GPs relate the opening dimensions to the retained coefficients in each section family. The training database contains the 12 combinations of *α* ∈ {0.30, 0.45, 0.60} and *β* ∈ {0.30, 0.45, 0.60, 0.75}. Inputs are scaled by the stated domain limits and outputs by training-fold statistics. A squared-exponential kernel with automatic relevance determination is adopted,

(25) kξ,ξ′=σf2 exp−12∑j=1nξξj−ξj′2lj2,

where *σ_f_* is the signal amplitude, $l_{j}$ are the characteristic length scales, and *σ_n_* is the regularization parameter used in Equation (26). For a new geometry **ξ**_∗_, the predicted coefficient is

(26) $${\hat{a}}_{r}\left(\xi_{*}\right)=k_{*}^{T}{\left(K_{\xi }+\sigma_{n}^{2}I\right)}^{-1}a_{r}.$$

The squared-exponential ARD kernel is used with one regularization term, 10^−10^ after output standardization. The two inputs are mapped by (*α* − 0.30)/0.30 and (*β* − 0.30)/0.45. Each output is standardized using only the current training set. Log length scales and log signal amplitude are fitted by marginal likelihood, bounded between −4 and 4, with three deterministic initializations. The linear correction surrogate is first evaluated using a fixed combined POD basis. A second leave-one-out calculation excludes the tested section–geometry snapshot before rebuilding the combined POD basis from the remaining 23 matrices. Regression uses only the remaining 11 samples of that section family. For the logarithmic surrogate introduced below, the basis and GPR are both fitted separately to the 11 training geometries in each fold. Fourteen additional off-grid shell evaluations are reserved for independent testing and never used for fitting or choosing the retained rank.

GPR places a Gaussian prior on a function evaluated at any finite set of inputs; it does not require a Gaussian histogram of the deterministic POD coefficients [40]. The squared-exponential ARD kernel encodes smooth variation and different length scales for the two geometry parameters. Smoothness is a modeling assumption appropriate to regular geometric changes, not a guarantee across remeshing or topology changes. POD coefficients need not be monotone. Rougher kernels or other surrogates may be appropriate after enlarging the domain, but no comparative evidence is claimed here.

For an unseen opening geometry, the predicted correction vector is reconstructed as

(27) $$\hat{d}\left(\xi_{*}\right)=U_{R}\hat{a}\left(\xi_{*}\right).$$

After applying the inverse symmetric-vectorization operator, the dimensionless element stiffness is obtained from

(28) $${\hat{\overset{‾}{K}}}_{e}\left(\xi_{*}\right)={\overset{‾}{K}}_{e}^{0}+{\mathrm{svec}}^{-1}\left[\hat{d}\left(\xi_{*}\right)\right],$$

and the dimensional matrix used in structural calculations follows as

(29) $${\hat{K}}_{e}=EA_{0}L_{*}S_{e}^{-T}{\hat{\overset{‾}{K}}}_{e}S_{e}^{-1},$$

Symmetric vectorization preserves symmetry but does not guarantee positive stiffness. Admissibility is checked on the complete dimensionless free element: the six analytical rigid-body motions must remain in its nullspace, and the stiffness restricted to their eight-dimensional orthogonal complement must be positive definite. The relative rigid-motion tolerance is 10^−8^ and the minimum dimensionless deformational eigenvalue must exceed 10^−8^ in the implementation. An inadmissible prediction is rejected and recomputed directly from the shell model. The linear three-mode surrogate is retained as a diagnostic baseline; the following logarithmic representation is used for the end-to-end predictive example.

Let **R** contain the six dimensionless rigid-body element motions and let **Q** ∈ ℝ^14×8^ have orthonormal columns spanning ker(**R***^T^*). Define the intact deformational stiffness and its Cholesky factor by $Q^{T}{\overset{‾}{K}}_{e}^{0}Q=L_{0}L_{0}^{T}$. For each section family, the stiffness is represented by

(29a) $$H=L_{0}^{-1}Q^{T}{\overset{‾}{K}}_{e}QL_{0}^{-T},\;Z=\log H,\;z=\mathrm{svec}\left(Z\right)\in R^{36},$$

Uncentered POD and separate scalar GPs are applied to the logarithmic vectors, using a 99.99% snapshot-energy criterion and a separate basis for C and Z. The predicted symmetric logarithm $\hat{Z}$ is mapped back as

(29b) $${\hat{\overset{‾}{K}}}_{e}=QL_{0}\exp\left(\hat{Z}\right)L_{0}^{T}Q^{T}.$$

The exponential of a real symmetric matrix is positive definite, and multiplication by **Q** preserves the specified rigid-body nullspace. Reference-energy scaling also gives weak deformation directions appropriate weight before compression. This uses the established log-Euclidean construction for positive-definite matrices [43], rather than eigenvalue clipping of an indefinite prediction. The POD energy criterion in this transformed space is distinct from the 99.9% criterion for the original correction matrices. Predictions are restricted to 0.30 ≤ *α* ≤ 0.60 and 0.30 ≤ *β* ≤ 0.75; other geometries require direct evaluation. The relative energy error is additionally assessed over every deformational state as max*_i_*|*λ_i_* − 1|, where *λ_i_* are the generalized eigenvalues of the predicted and directly computed eight-dimensional stiffness matrices. The surrogate is strictly interpolation-only: out-of-domain inputs are rejected before prediction and require direct shell condensation.

The reduced matrices are subsequently assembled in the same manner as conventional finite elements. Adjacent perforated segments share the seven generalized coordinates at their common intact cross-section, including the warping amplitude *γ_ω_*. For element *e*, let **A***_e_* denote the Boolean connectivity matrix and **T***_e_* the local-to-global coordinate transformation. The global reduced stiffness is

(30) $$K_{G}=\sum_{e=1}^{n_{e}}A_{e}^{T}T_{e}^{T}K_{e}^{\left(e\right)}T_{e}A_{e}.$$

**Figure 5:**
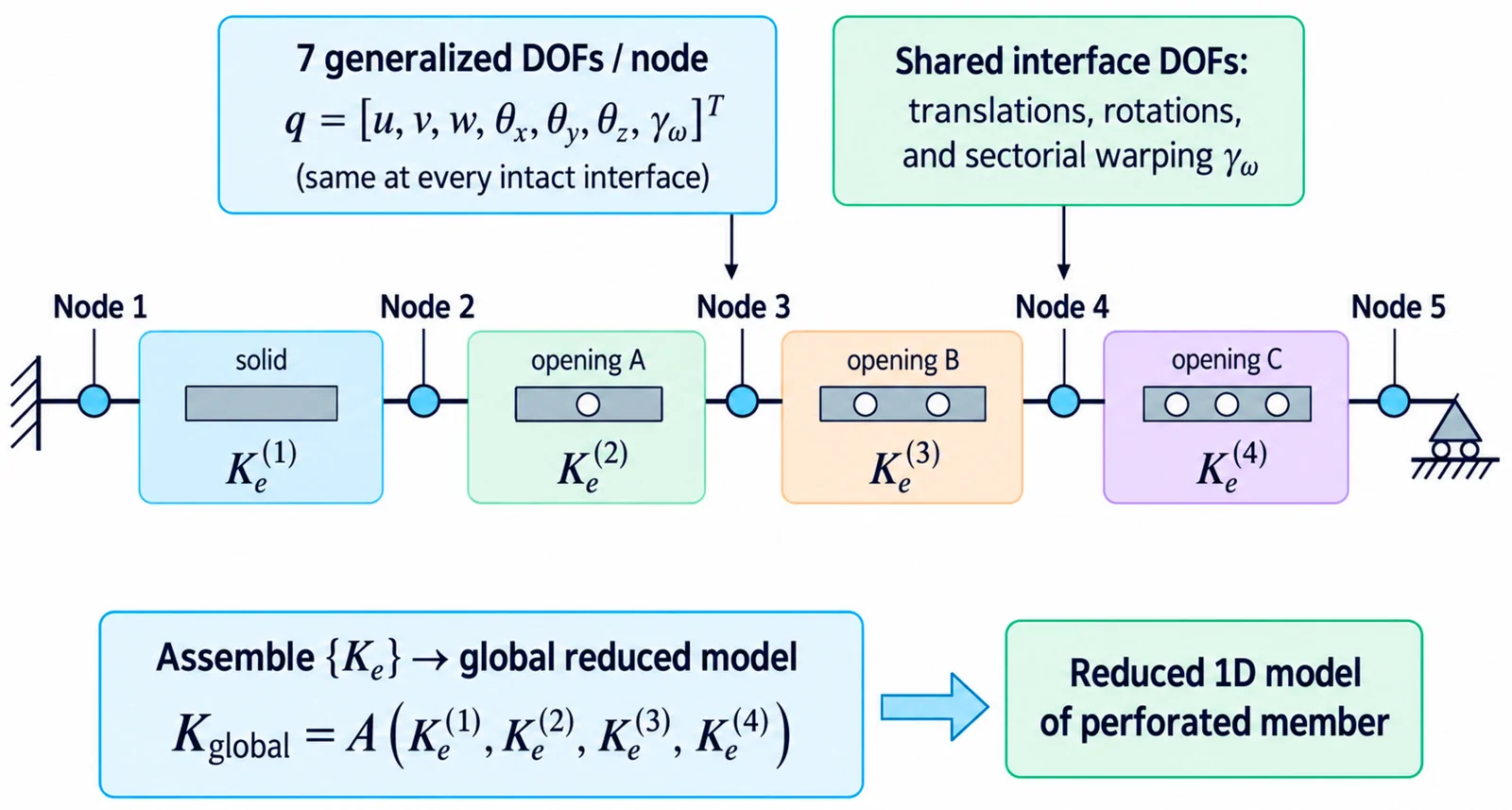
Assembly of a perforated member from reduced 14 × 14 elements. Each intact interface carries seven generalized degrees of freedom; the sectorial warping amplitude is shared between adjacent elements in the same manner as the classical translations and rotations.

For straight C- and Z-members aligned with the global axis, **T***_e_* reduces to the identity matrix. The generalized force vector work-conjugate to the seven nodal coordinates is written as

(31) $$p={\left[N,V_{y},V_{z},T,M_{y},M_{z},B\right]}^{T},$$

where *B* is the generalized bimoment associated with *γ_ω_*. Free warping at an external end is a natural boundary condition corresponding to *B* = 0, whereas fully restrained warping is imposed by *γ_ω_* = 0. Figure 5 shows the assembly of a long perforated member from geometry-dependent reduced elements and illustrates the treatment of the shared warping degree of freedom.

The independent warping coordinate has a variational interpretation. With compatible generalized coordinates collected in **Q**, the reduced potential is $\Pi \left(Q\right)=1/2\sum_{e}q_{e}^{T}K_{e}q_{e}-Q^{T}F$, where **q***_e_* = **T***_e_***A***_e_***Q**. Stationarity gives Equation (30). At an interior interface shared by two elements, variation of the common warping amplitude gives $B_{R}^{\left(e\right)}+B_{L}^{\left(e+1\right)}=B_{\mathrm{ext}}$ in a consistent work-conjugate sign convention; in the absence of an applied bimoment the sum is zero. Sharing the amplitude therefore enforces continuity of the retained axial-warping trace and automatically produces generalized bimoment equilibrium. It does not impose a continuous classical twist-derivative field inside the segments, where the deformation is represented by the shell equilibrium solution.

This variational consistency applies to the chosen reduced model. It does not restore shell-rotation continuity that has been removed before assembly. Independently minimizing the rotational energy in two neighboring segments allows different minimizing rotations on the two sides; minimizing with a common rotation imposes an additional compatibility condition and cannot give a lower constrained energy. Exact substructuring of a continuous shell model would retain and assemble those shared rotations, or otherwise enforce their compatibility before elimination. The present translation-only assembly must therefore be verified against the continuous shell member rather than described as an exact consequence of local static condensation.

Verification separates four questions. Same-mesh tests examine algebraic reduction and reaction recovery; continuous shell members assess interface truncation and rotational relaxation; classical unperforated Vlasov solutions provide an independent torsion and warping reference; and nested plus independent off-grid tests assess the complete surrogate. Table 3 summarizes this validation hierarchy. In the member reference, only the two physical end traces are constrained as described below; interior cross-sections retain all shell degrees of freedom. Experimental stiffness measurements were not part of this study, so numerical and analytical agreement is not described as physical validation of manufactured members.

For quantitative comparison of surrogate-predicted and directly condensed stiffness corrections, the relative Frobenius error is defined as

(32) $$e_{\Delta K}=\frac{{∥{\hat{\Delta \bar{K}}}_{e}-\Delta {\bar{K}}_{e}∥}_{F}}{{∥\Delta {\bar{K}}_{e}∥}_{F}}.$$

Because matrix agreement alone does not guarantee identical structural behavior, an energy-based error is evaluated for prescribed generalized displacement states,

(33) $$e_{U}=\frac{\left|{\hat{U}}_{e}-U_{e}\right|}{\left|U_{e}\right|},\;\;U_{e}=\frac{1}{2}q_{e}^{T}K_{e}q_{e}.$$

For comparison with the full shell member, translational shell displacements extracted at selected intact cross-sections are projected onto the seven-dimensional interface basis using a weighted least-squares projection,

(34) $$q_{\mathrm{FE}}={\left(\Phi^{T}W\Phi \right)}^{-1}\Phi^{T}Wu_{\Gamma }^{\mathrm{FE}}.$$

The matrix **W** contains positive nodal tributary-area weights. Equation (34) extracts generalized coordinates by post-processing and does not constrain the reference shell solution. A combined norm of translations, rotations, and warping amplitudes requires dimensional scaling. In Equation (35), the stacked generalized vectors are therefore written in dimensionless form using the block-diagonal extension S of **S**_7_. The displacement and twist profile errors reported separately in The separately reported displacement and twist profile errors compare like quantities and are distinct from a single norm combining all seven coordinates.

(35) $$e_{q}=\frac{∥{\overset{‾}{q}}_{\mathrm{red}}-{\overset{‾}{q}}_{\mathrm{FE}}{∥}_{2}}{∥{\overset{‾}{q}}_{\mathrm{FE}}{∥}_{2}},\;\;\overset{‾}{q}=S^{-1}q.$$

For a single segment, the Schur complement is exact for the stated retained variables and zero loads on the eliminated variables, up to linear-solver accuracy. Approximation enters through the seven-mode interface restriction, POD truncation, and coefficient regression. There is a further distinction at member level: eliminating shell rotations independently in neighboring segments does not enforce their continuity across the common cut. The assembled translation-only model therefore differs from a continuous shell model both through its interface basis and through this rotational relaxation. The member-level comparisons assess their combined effect; they should not be interpreted as isolating projection error alone.

## 3. Results

The results were organized to assess the proposed framework at four successive levels: (i) numerical consistency of the shell model and the interface reduction, (ii) ability of the reduced element to reproduce the stiffness of individual perforated segments, including warping effects, (iii) low-dimensional representation and prediction of the opening-induced stiffness correction, and (iv) accuracy and computational efficiency of structural models assembled from the reduced elements. The same sequence was applied to both C- and Z-sections so that the influence of cross-sectional asymmetry and stiffness coupling could be assessed consistently.

### 3.1. Numerical Convergence and Accuracy of the Reduced Element

**Figure 6:**
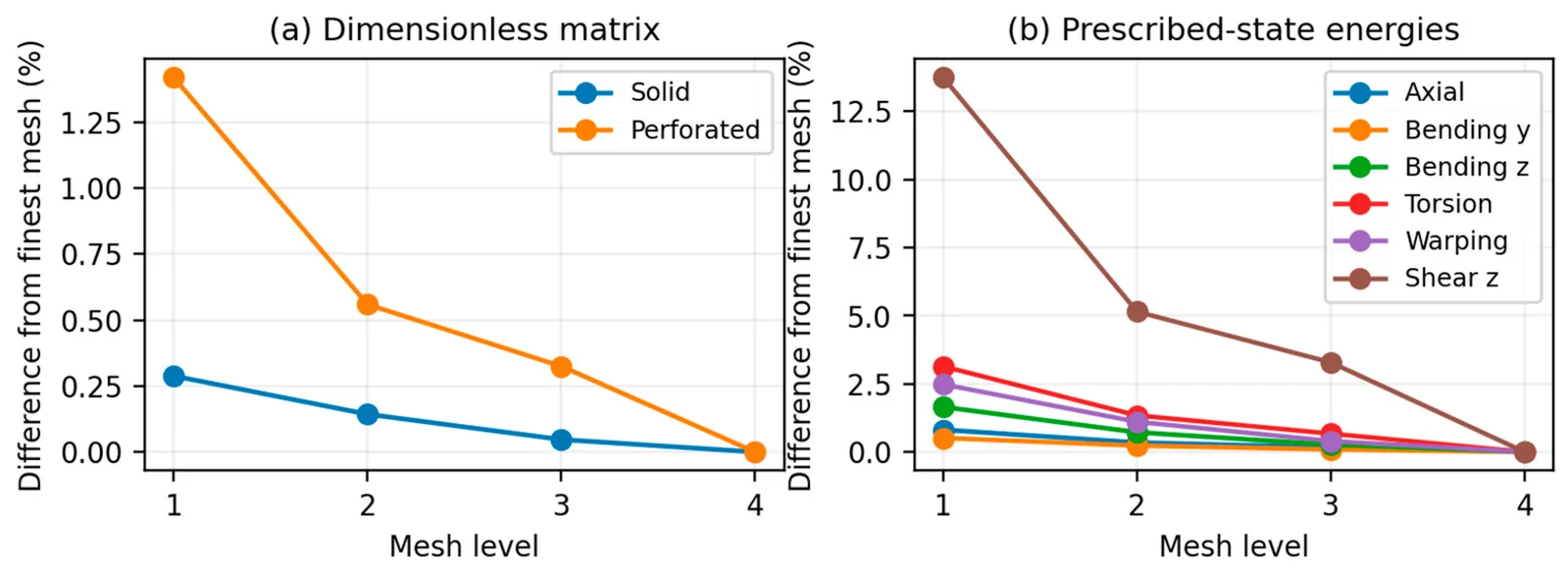
Mesh-convergence characteristics: (**a**) relative error of the complete reduced stiffness matrix for the solid and representative perforated C-section; (**b**) convergence of selected generalized energetic stiffness measures for the representative perforated C-section.

Four progressively refined meshes were evaluated using the same mesh-independent sectorial basis, dimensionless scaling, and prescribed generalized displacement states. The finest mesh is the numerical reference. Figure 6 and Table 4 report complete-matrix errors and component-energy differences. Both measures are required because the matrix norm is dominated by its larger entries.

At the adopted third mesh, the complete-matrix differences from the finest solution are 0.047% for the intact segment and 0.325% for the perforated segment. For the latter, the axial, major/minor bending, torsion, and warping energy differences are 0.172%, 0.094%/0.267%, 0.673%, and 0.400%, respectively. The shear-related state is less converged, with a 3.288% difference; the small complete-matrix error must therefore not be interpreted as sub-percent convergence of every stiffness component. The third mesh is used consistently for the database and member comparisons. No convergence claim is made for corner stresses, which are not outputs of the reduced model.

Four interface variants were compared on a six-segment C-section member with identical openings, *α* = 0.60 and *β* = 0.5333. The common continuous-shell reference has the free-warping end conditions specified below. The applied actions are *N* = 100 N, *M_y_* = 1 N m, *M_z_* = 1 N m, *T* = 1 N m, and (*M_y_*,*T*) = (1,0.5) N m. Table 5 compares strain energies under these fixed generalized loads, *U* = 1/2**F***^T^***Q**; this is a compliance comparison, distinct from the prescribed-displacement energy in Equation (33). The mean is the arithmetic mean of the five absolute relative errors.

The axial and bending compliance errors are below 0.65% for these variants. Omitting sectorial warping, however, produces a torsional energy error of 99.870%. The B6 trace forces the axial warping amplitude to vanish at successive interfaces and therefore suppresses the dominant torsional compliance of this open section.

For the B7 trace, retaining only translations gives a torsional energy error of 0.102%; tying retained shell rotations to rigid-section rotations instead gives 82.142%. This concerns the final constrained trial space, not the information content of the unprojected Schur complement. An alternative six-DOF treatment, in which the interface rotations are determined by energy-minimizing harmonic lifting for each translational trace, reproduces the translation-only matrix to within 1.09 × 10^−13^ in relative dimensionless norm across the four solid/perforated C/Z checks. Thus retaining rotations is not intrinsically inaccurate: the imposed rotational map is decisive. This two-end optimal lifting is not itself a proof of compatible rotations after independent segment assembly.

**Figure 7:**
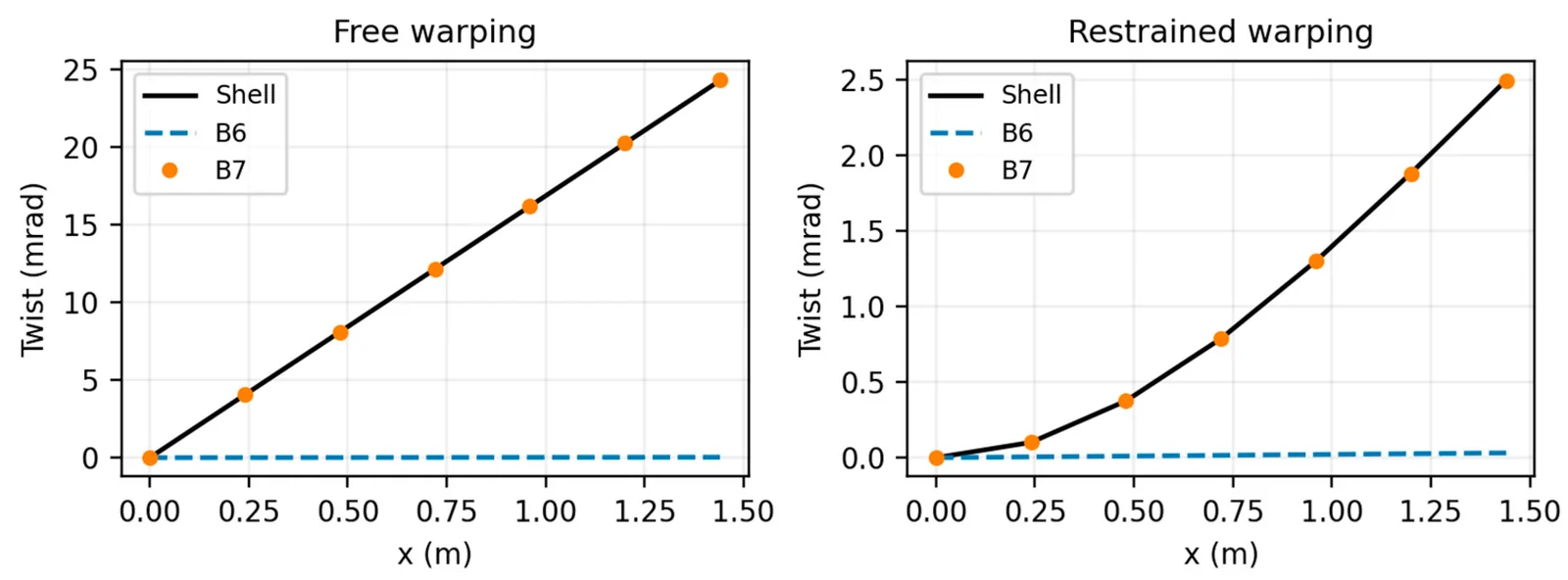
Influence of warping enrichment on torsional response: (**left**) free-warping case; (**right**) restrained-warping case. The full shell solution is compared with the six-DOF and seven-DOF reduced formulations.

Figure 7 considers six identical perforated C-section segments, each *L_e_* = 0.240 m, giving *L* = 1.44 m, under *T* = 1 N m. The opening dimensions are *α* = 0.60 and *β* = 0.5333. The load scale was reduced from the original presentation so that the displayed rotations remain small; all comparisons are based on the initial linear stiffness.

The shell boundary conditions are imposed by exact elimination of multipoint constraints at the two physical ends: **u***_ΓL_* = **Φ***_L_***q***_L_* and **u***_ΓR_* = **Φ***_R_***q***_R_*, using translations only. At the left end the six classical generalized coordinates are zero. Free warping leaves *γ_ωL_* unknown with zero applied bimoment; restrained warping additionally sets *γ_ωL_* = 0, so all left-end translations then vanish. At the right end all seven generalized coordinates remain unknown and the applied generalized load vector has the specified torque or force components, with zero bimoment. Torque is conjugate to *θ_xR_* through virtual work; its nodal reactions are obtained from the end transformation, rather than an arbitrary nodal couple. All shell nodal rotations at both physical ends remain unconstrained, and cut sections inside the continuous member are completely unconstrained. These are idealized modal end attachments, not a claim to reproduce a particular experimental clamp. The B7 member uses the same generalized end conditions.

With free warping, the B6 and B7 twist-profile errors relative to the continuous shell member are 99.870% and 0.102%, respectively. With restrained warping, they are 98.596% and 0.253%. These figures apply to the explicitly stated modal end attachments and the current common discretization. They demonstrate the importance of the sectorial coordinate for the tested member, without providing a universal error bound for every support or perforation pattern.

### 3.2. Reduced Stiffness Matrices and Opening-Induced Stiffness Modification

The influence of a web opening was next examined directly at the matrix level. The reduced stiffness matrix of each perforated segment was compared with the corresponding unperforated reference through the dimensionless correction $\Delta {\bar{K}}_{e}={\bar{K}}_{e}-{\bar{K}}_{e}^{0}.$

For a centered rectangular opening with *h_o_*/*h* = 0.60 and *b_o_*/*h* = 0.533, the relative Frobenius norm of the stiffness correction reached 28.45% for the C-section and 28.06% for the Z-section. The correction was clearly non-uniform across the matrix, demonstrating that a perforation cannot be represented accurately by a single scalar reduction factor. Instead, the opening modifies individual stiffness contributions and generalized couplings to different degrees.

The global magnitude of the opening-induced correction for the representative centered rectangular opening is summarized in Table 6.

**Figure 8:**
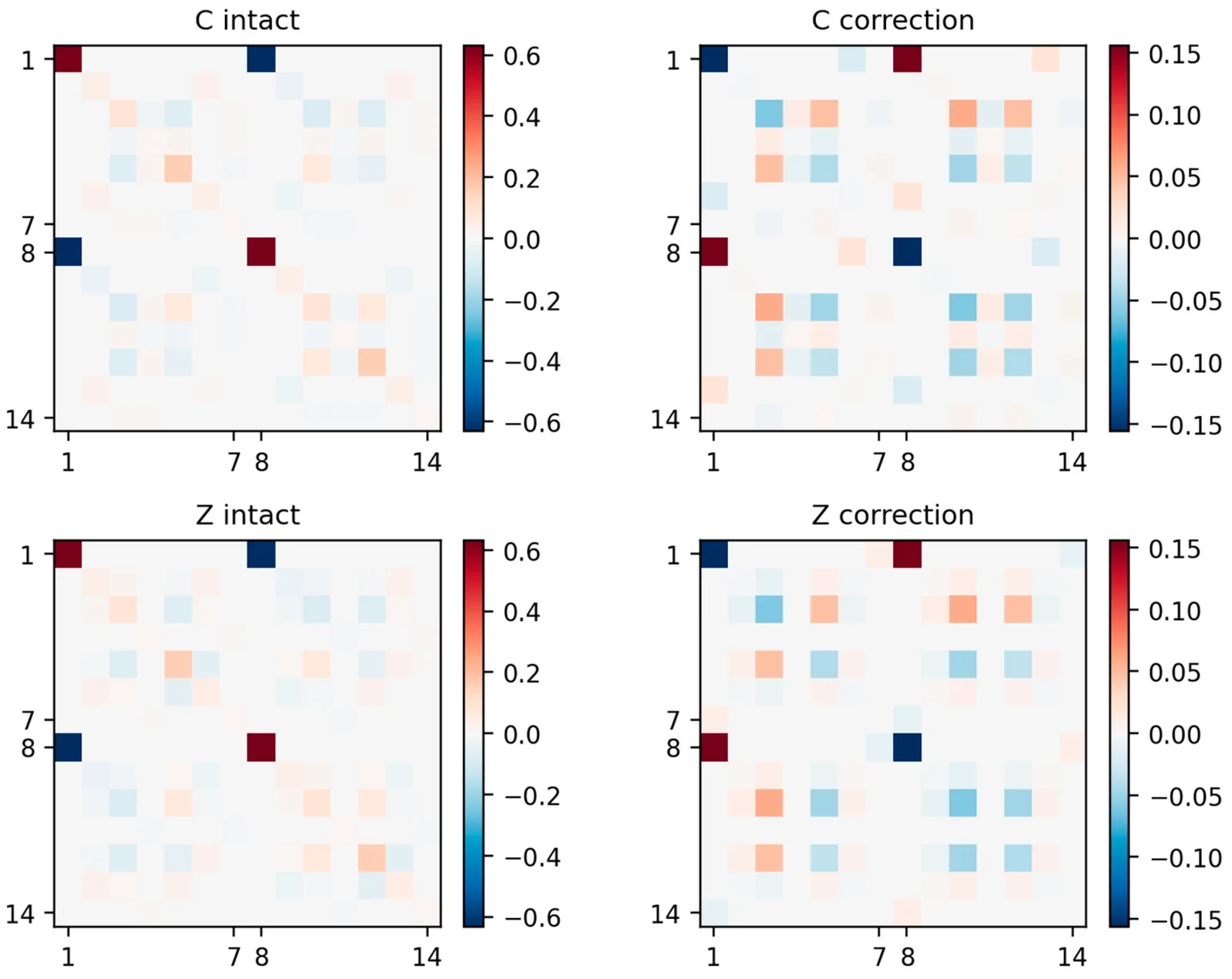
Dimensionless reduced stiffness matrices and opening-induced corrections: (**upper left**) ${\bar{K}}_{e}^{0}$ for the C-section; (**upper right**) $\Delta {\bar{K}}_{e}$ for a representative perforated C-section; (**lower left**) ${\bar{K}}_{e}^{0}$ for the Z-section; (**lower right**) $\Delta {\bar{K}}_{e}$ for the corresponding perforated Z-section.

For the representative centered rectangular opening, the global correction magnitude was almost identical for both section families, reaching 28.45% for the C-section and 28.06% for the Z-section, although Figure 8 shows that the distribution of individual matrix contributions differed between the two cross-sections.

**Figure 9:**
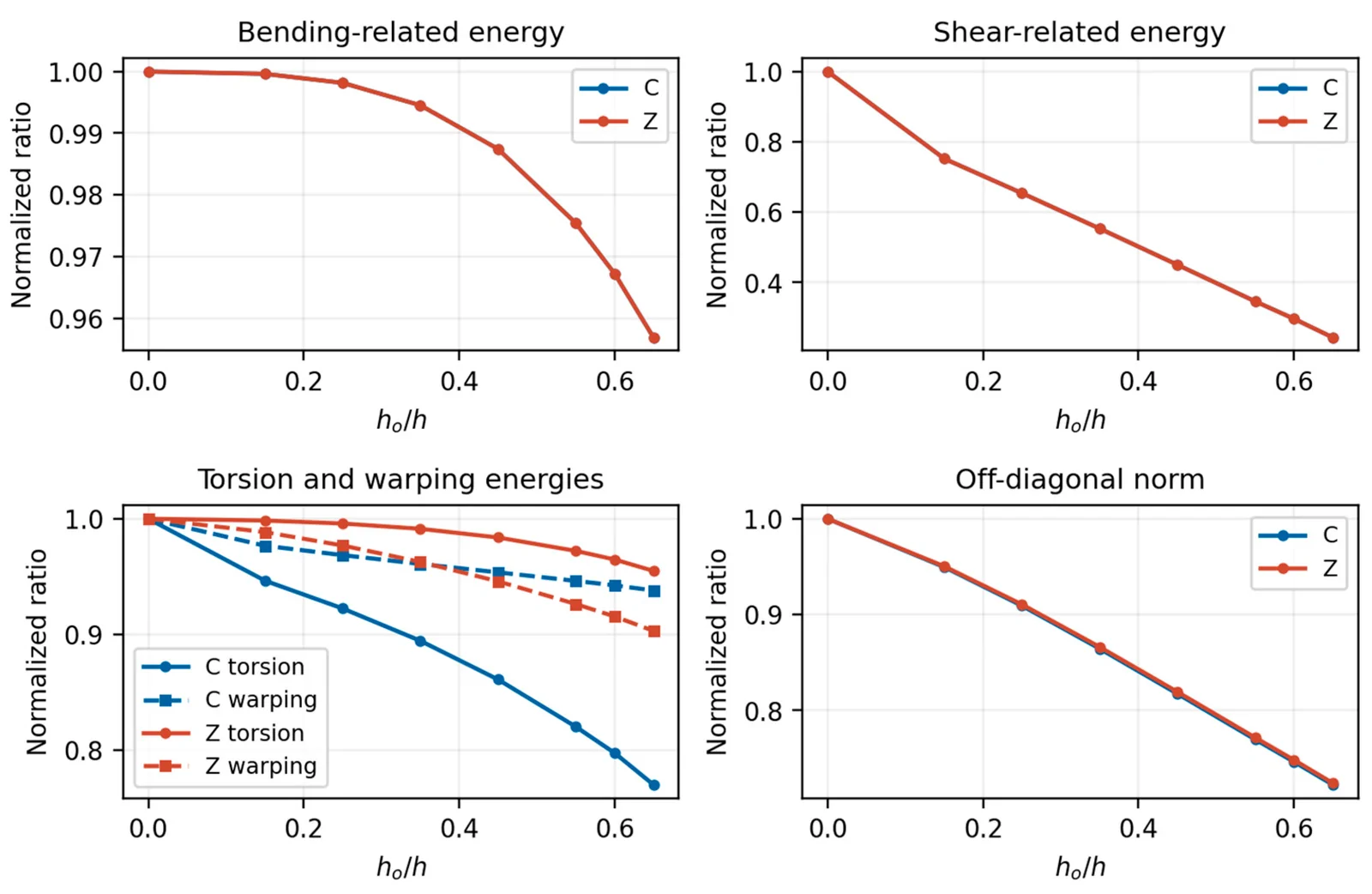
Effect of normalized opening height on the reduced stiffness response of C- and Z-sections at *b_o_*/*h* = 0.533: (**upper left**) major-axis bending-related stiffness; (**upper right**) transverse-shear-related stiffness; (**lower left**) torsional and warping-related stiffness; (**lower right**) normalized off-diagonal coupling norm.

**Figure 10:**
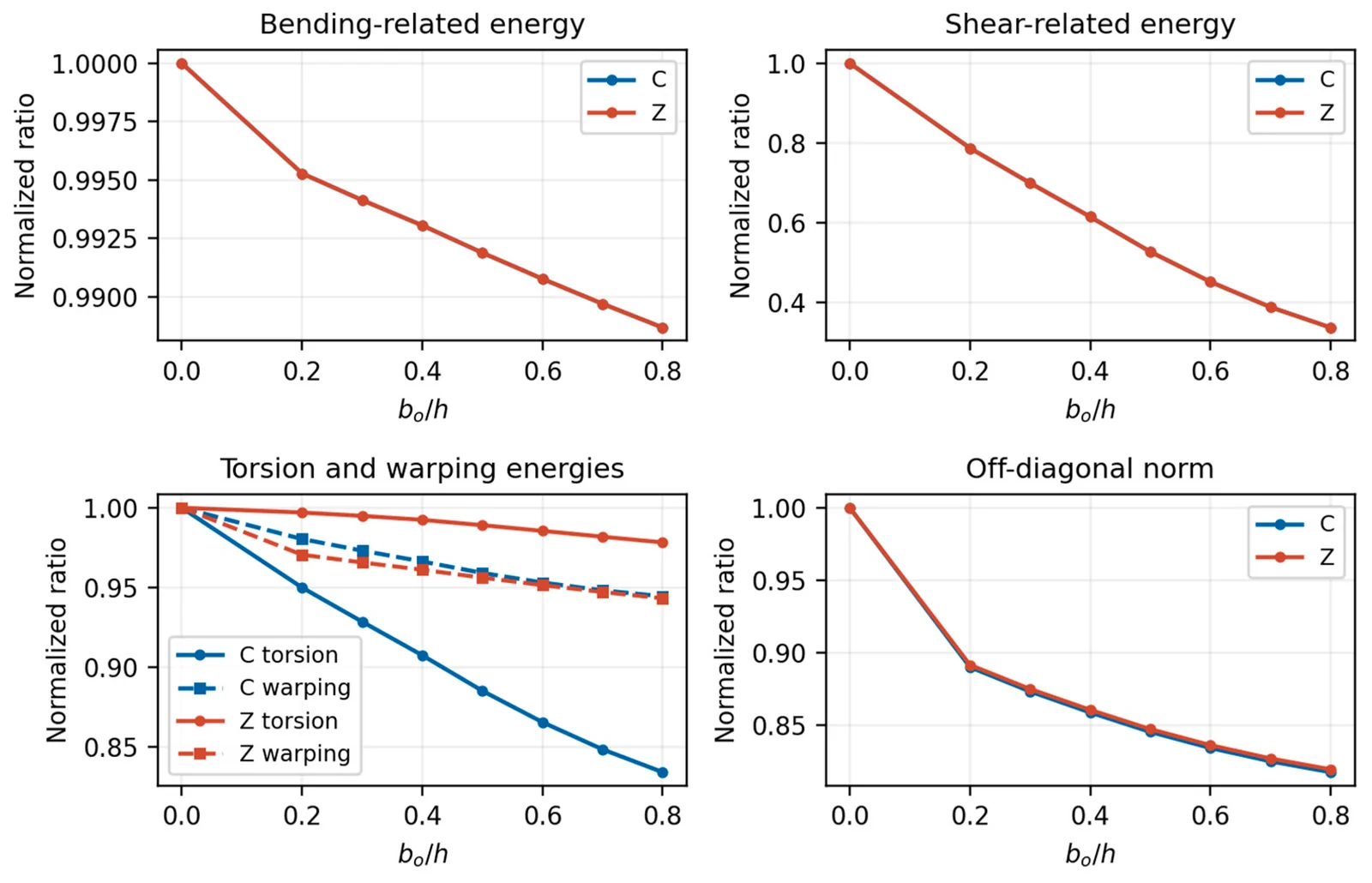
Effect of normalized opening length on the reduced stiffness response of C- and Z-sections at *h_o_*/*h* = 0.40: (**upper left**) major-axis bending-related stiffness; (**upper right**) transverse-shear-related stiffness; (**lower left**) torsional and warping-related stiffness; (**lower right**) normalized off-diagonal coupling norm.

Every ordinate in Figure 9 and Figure 10 is reproducible from a specified dimensionless state. Let **e***_i_* denote the *i* th unit vector in the 14-coordinate ordering of Equation (11), and let *λ* = *L_e_*/*h*. The bending-related state is **d***_b_* = −*λ*^2^**e**_10_/2 + *λ***e**_12_, the shear-related state is **d***_s_* = **e**_10_, the torsion-related state is **d***_t_* = **e**_11_, and the warping-related state is **d***_w_* = **e**_14_. The plotted ratio is $R_{d}=d^{T}{\overset{‾}{K}}_{e}d/\left(d^{T}{\overset{‾}{K}}_{e}^{0}d\right)$. These are constrained-state energy ratios, not independently identified sectional properties such as *GJ* or *EI_ω_*. The coupling ratio is the Frobenius norm of all off-diagonal entries divided by its intact counterpart, with the same dimensionless ordering. Table 6 instead reports $100∥\Delta {\overset{‾}{K}}_{e}{∥}_{F}/∥{\overset{‾}{K}}_{e}^{0}{∥}_{F}$.

The sensitivity to opening height was then investigated for centered rectangular openings at constant *b_o_*/*h* = 0.533.

At *α* = 0.65, the shear-related ratio is approximately 0.244 for both section families, while the bending-related ratio remains approximately 0.957. The torsion-related ratios are 0.770 for C and 0.955 for Z. For the explicitly defined warping state, they are 0.938 and 0.903, respectively. The off-diagonal norm ratios are 0.723 and 0.724. The different torsional response reflects the different energy distribution and coupling of these geometries; it is not a general invariance of Z-section torsion.

A complementary analysis was performed by varying the longitudinal opening dimension while maintaining *h_o_*/*h* = 0.40.

At *β* = 0.80 and *α* = 0.40, the bending-related ratio is 0.989 and the shear-related ratio is 0.337 for both families. The torsion-related ratios are 0.834 for C and 0.978 for Z. Warping-related ratios are 0.944 and 0.943, and coupling-norm ratios are 0.817 and 0.819. The web opening therefore changes the shear-dominated state much more than the selected bending state, while torsional and warping measures remain sensitive to the exact state definition and section geometry.

### 3.3. Low-Dimensional Structure of the Stiffness Correction

**Figure 11:**
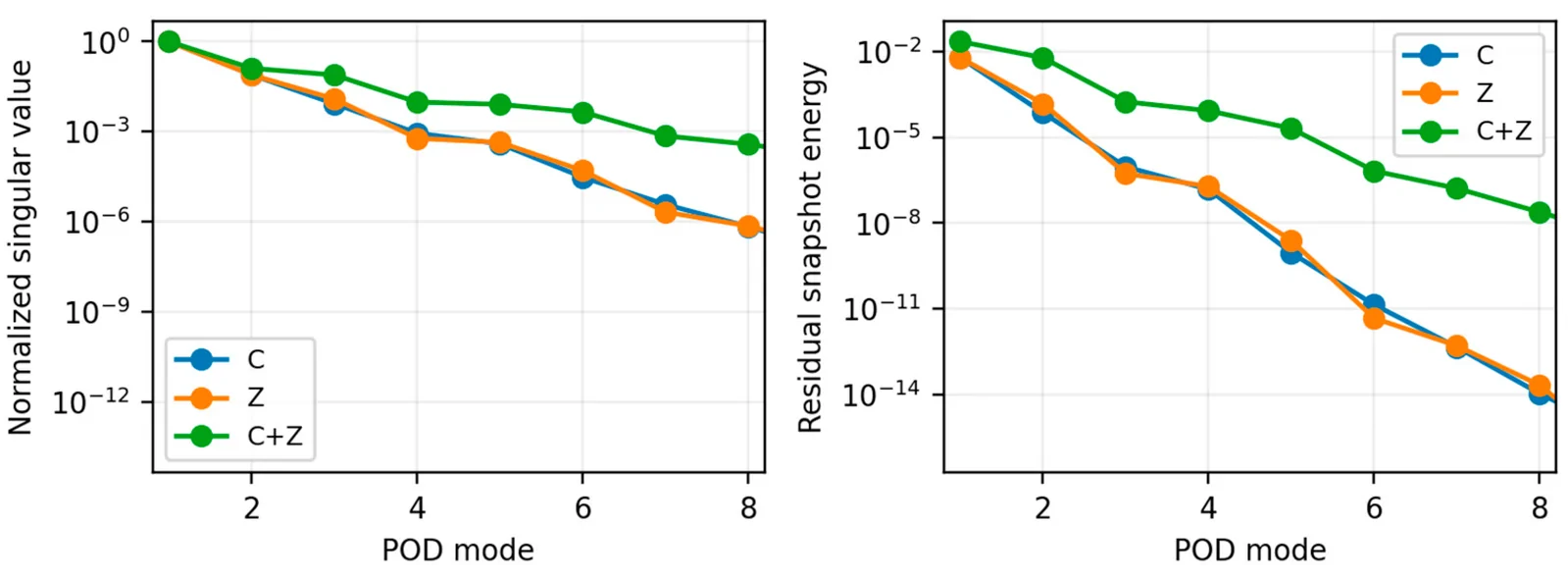
POD characteristics of the opening-correction database: (**left**) normalized singular values; (**right**) residual snapshot energy after retaining successive modes.

The correction database contains 12 rectangular-opening geometries per family, formed from *α* ∈ {0.30, 0.45, 0.60} and *β* ∈ {0.30, 0.45, 0.60, 0.75}. The combined POD therefore uses 24 dimensionless correction matrices. All section dimensions, material properties, and segment lengths remain fixed. Figure 11 shows the singular-value decay and residual snapshot energy; Table 7 summarizes the POD compression.

A pronounced singular-value decay was observed. For the C-section database, a single POD mode captured more than 99% of the total energy, while two modes exceeded 99.9%. The same behavior was obtained for the Z-section. When both section types were combined into a single database, one mode still represented more than 95% of the energy, two modes exceeded 99%, and only three modes were required to exceed 99.9%.

**Figure 12:**
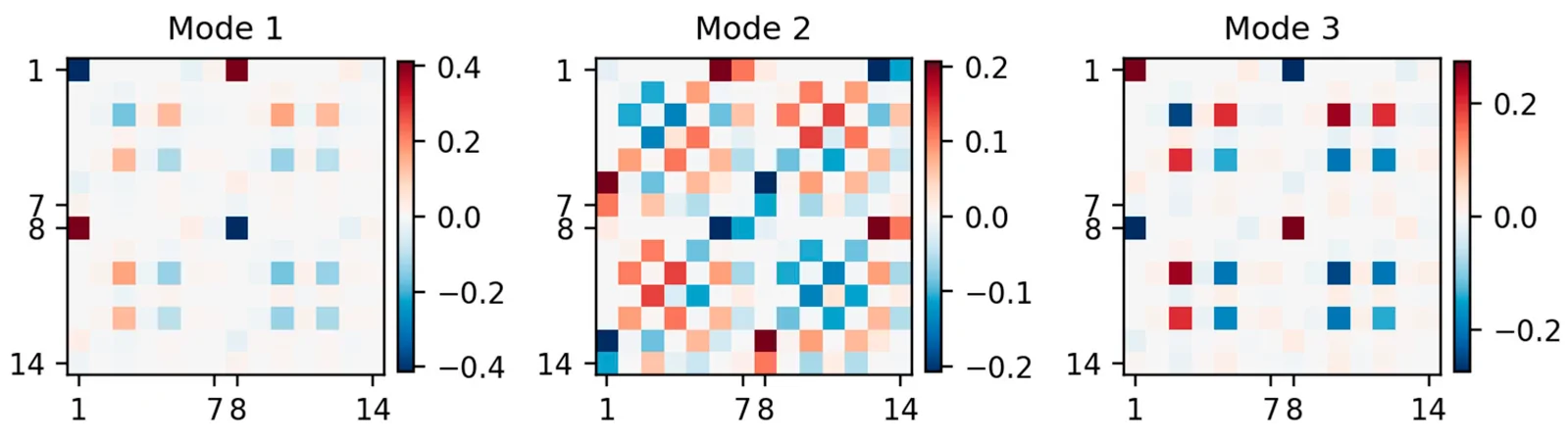
Leading POD modes of the dimensionless stiffness-correction matrix for the combined C+Z database: (**left**) ***M***_1_; (**middle**) ***M***_2_; (**right**) ***M***_3_.

At the 99.9% criterion, the mean correction-matrix reconstruction errors are 0.888% for C, 1.300% for Z, and 1.468% for the combined database; the corresponding maxima are 2.069%, 3.092%, and 3.004%. The first three combined modes are shown in Figure 12. These Frobenius errors describe representation of the correction and do not by themselves establish energetic admissibility.

**Figure 13:**
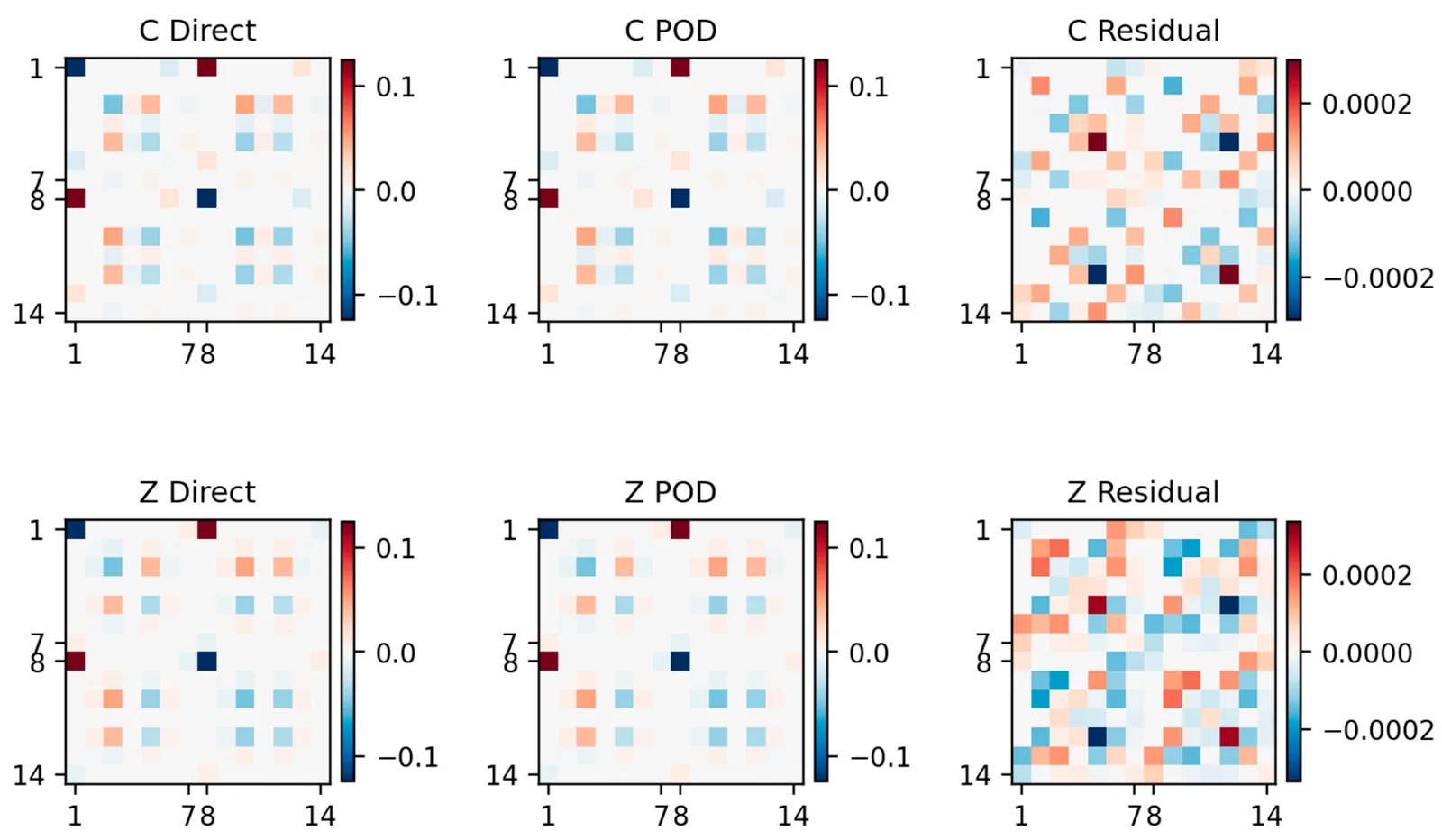
Comparison between directly condensed and POD-reconstructed stiffness corrections for representative perforated segments: direct result, reconstructed result, and residual matrix in the left, middle, and right columns, respectively; C-section results in the upper row and Z-section results in the lower row.

The first combined mode accounts for 97.799% of the snapshot energy, followed by 1.602% and 0.581%; the cumulative value is 99.9826%. Thus the matrix perturbations are strongly correlated in this parameter family. A low-rank description in this norm nevertheless can lose stiffness directions whose absolute contribution to the matrix norm is small. Figure 13 compares the directly condensed and reconstructed corrections and their residuals for both section families.

A local explanation of the low rank follows by expanding the correction vector around one geometry: **d**(*α*,*β*) ≈ **d**_0_ + **d**_,*α*_Δ*α* + **d**_,*β*_Δ*β*. To first order, two smoothly varying geometry inputs therefore generate an affine family lying in the span of at most three vectors. Shared symmetry and correlated changes in web-dominated deformation can further concentrate the snapshot energy. This is a local interpretation, not a proof that the full nonlinear parameter family has rank three. Symmetry reduces a 14 × 14 matrix to 105 independent entries, but symmetry or sparsity alone does not explain the observed POD rank. Moreover, the uncentered POD energy is a sum of squared dimensionless matrix norms, not a fraction of structural strain energy for every applied load.

The representational capability of this low-dimensional basis was additionally verified for an intermediate geometry not included in the POD snapshot grid, with *h_o_* / *h* = 0.50, *b_o_*/*h* = 0.533.

For the additional geometry *α* = 0.50, *β* = 0.5333, projection onto the combined three-mode basis gives correction errors of 0.319% for C and 0.428% for Z. This is a direct POD representation test; the shell correction at that geometry is projected onto an already fitted basis. It is distinguished from the independent GPR prediction reported below.

### 3.4. Surrogate Prediction of Geometry-Dependent Reduced Stiffness

**Figure 14:**
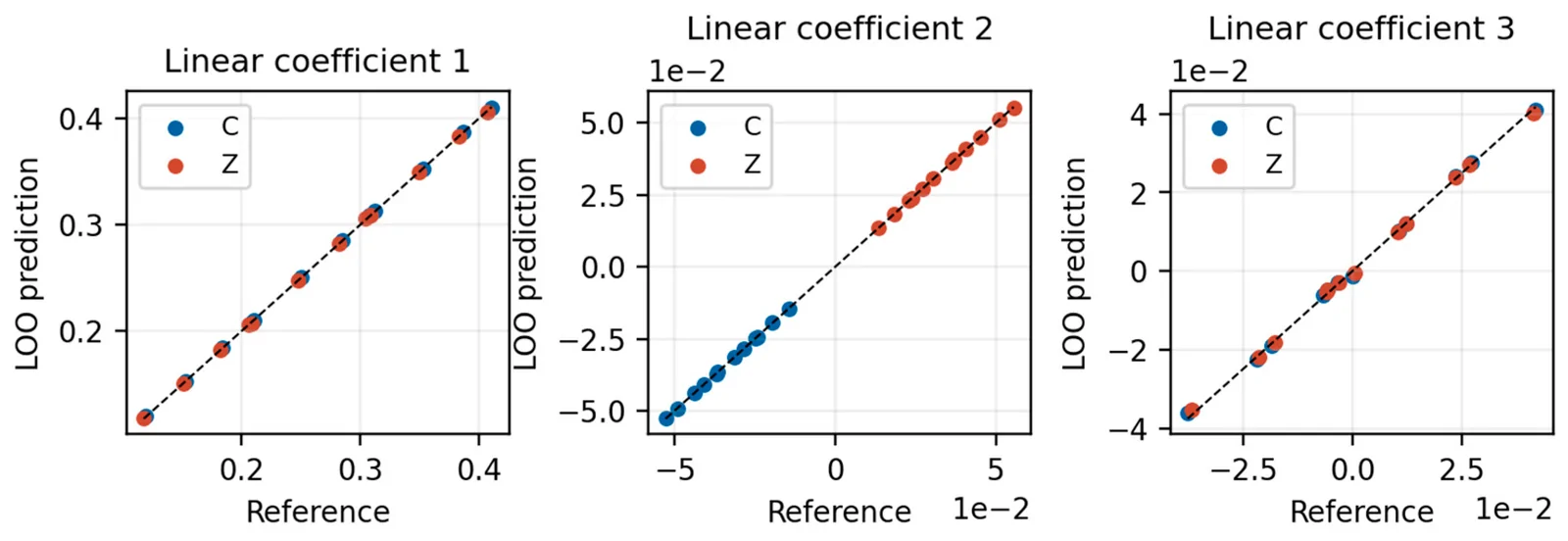
Leave-one-out surrogate predictions of the retained POD coefficients: predicted versus reference values for *a*_1_, *a*_2_, and *a*_3_ (left, middle, right panels, respectivelly). C- and Z-section samples are indicated separately; the dashed line represents perfect agreement.

Surrogate verification first examines the original linear three-mode correction model as a baseline, and then the logarithmic formulation of Equations (29a) and (29b) intended for member prediction. The two section families use separate scalar GPs. The baseline uses the combined matrix POD basis, whereas the logarithmic representation uses an energy-scaled basis fitted separately for each family. Figure 14 compares the leave-one-out coefficient predictions with their reference values.

For the fixed-basis linear baseline, the three coefficient *R*^2^ values are 0.999947, 0.999978, and 0.998806; range-normalized RMSE values are 0.220%, 0.156%, and 0.929%. These high coefficient scores do not establish accurate stiffness in the weak energetic directions. Table 8 reports the coefficients and correction errors, supplemented by full-element admissibility and member-response checks.

The linear baseline gives a pooled mean correction error of 1.517% and a maximum of 3.166% in fixed-basis leave-one-out validation. Rebuilding the combined POD basis within each fold increases these values to 1.718% and 3.214%. Despite these small matrix errors, 12 of 24 fixed-basis predictions and 8 of 14 independent off-grid predictions fail the positive-stiffness screen. The raw error metrics include these failed predictions; they are not selectively removed.

**Figure 15:**
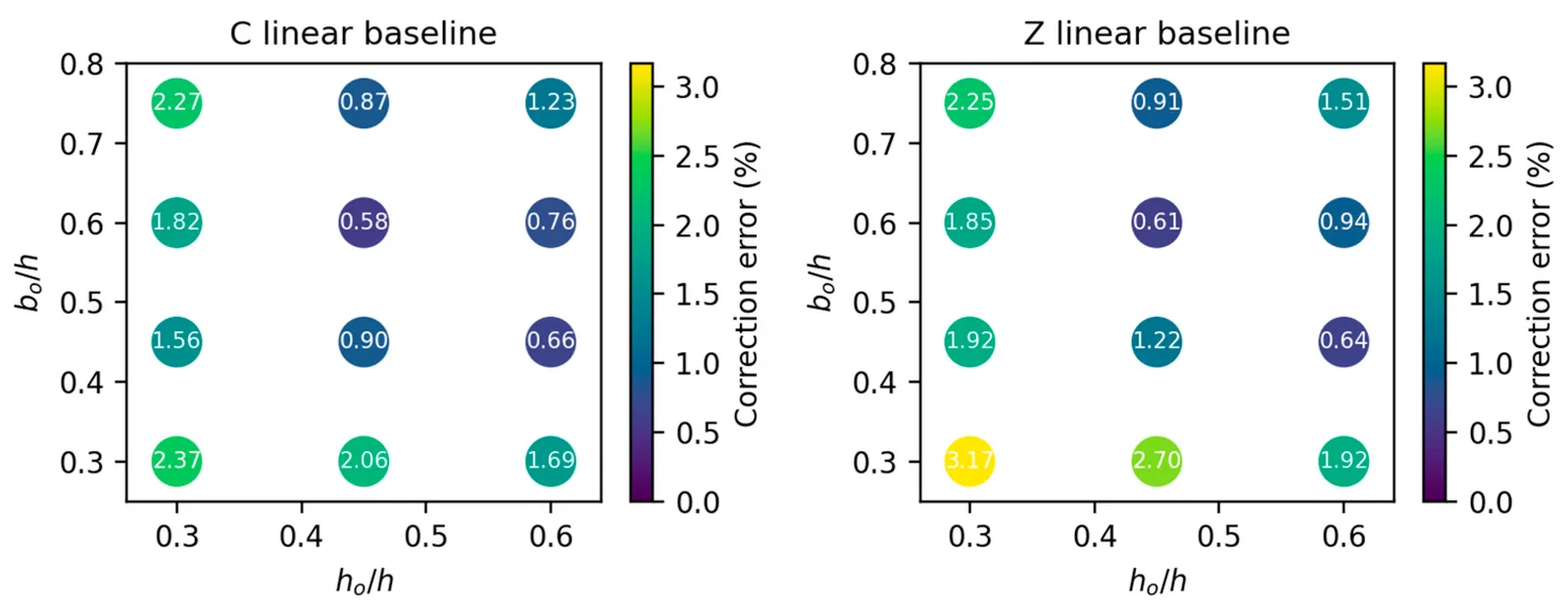
Fixed-basis leave-one-out errors of the linear correction surrogate at the sampled opening-height and opening-length pairs: (**left**) C-section; (**right**) Z-section. Values are correction-matrix errors in percent and include inadmissible predictions.

Figure 15 displays the pooled leave-one-out errors at the sampled parameter combinations. It is not an independently validated error bound between these combinations.

Figure 15 shows only the fixed-basis linear baseline. Its failure in weak modes is consequential: positivity screening with direct recomputation of rejected elements still leaves a four-segment off-grid test with displacement/twist errors of 28.825%/26.401% for C and 7.538%/19.028% for Z, because positive definiteness alone is not an accuracy certificate. These failures motivate the energy-scaled logarithmic model. With a 99.99% transformed snapshot-energy threshold, this model also retains three coefficients per section family. Fully nested leave-one-out validation gives pooled mean/maximum correction errors of 0.231%/0.911%. The 14 independent off-grid cases give 2.055%/2.932%, with a maximum complete-matrix error of 0.594% and maximum error over all deformational energies of 3.646%. All predictions preserve positive deformational stiffness. The independent errors are larger than the leave-one-out errors, which illustrates why the latter alone are insufficient.

### 3.5. Structural Assembly and Validation Against Full Shell Models

**Figure 16:**
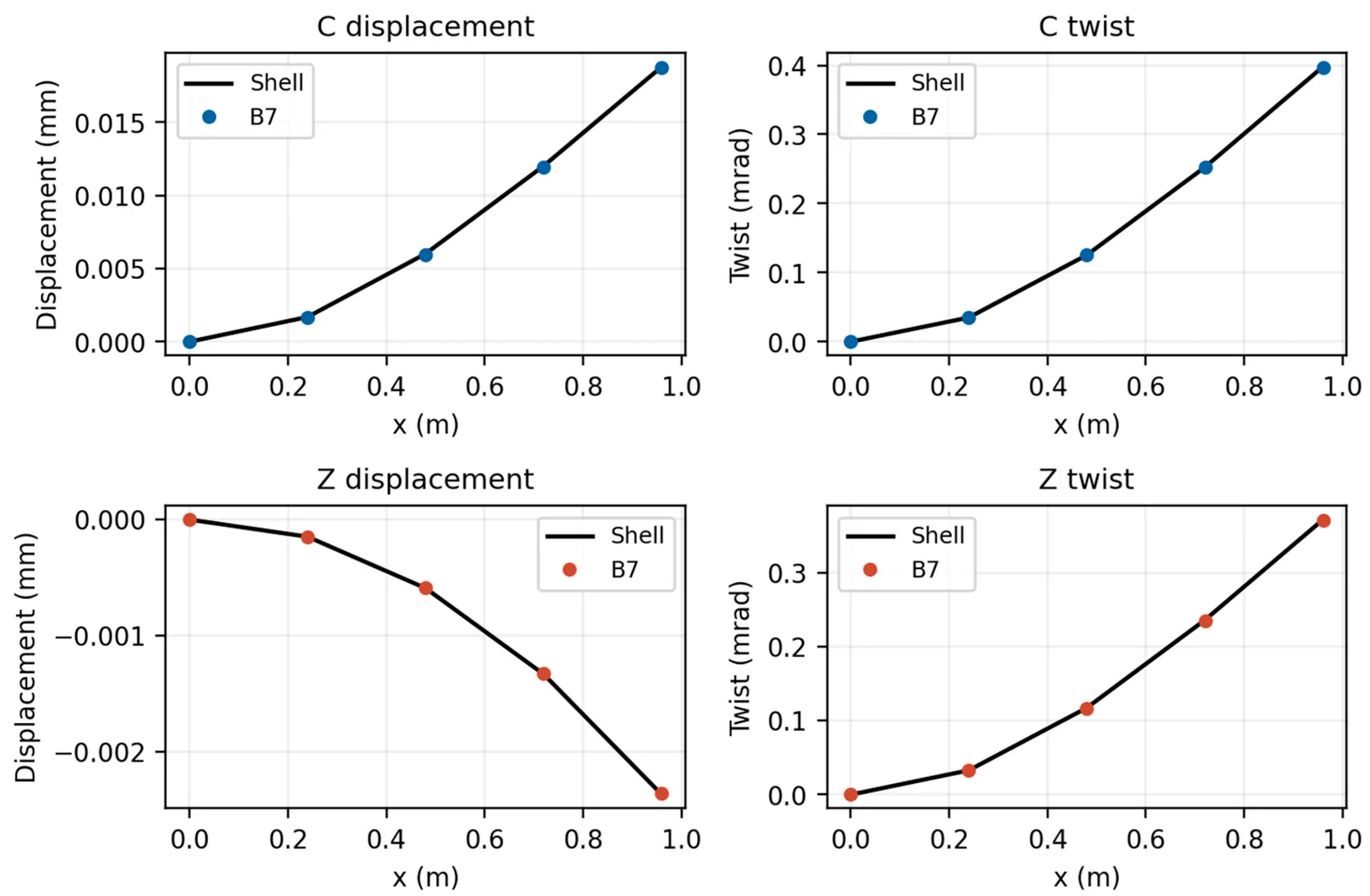
Validation of the assembled seven-DOF reduced model against the full shell model for a member containing four identical perforated segments under combined bending and torsion: (**upper left**) C-section transverse displacement; (**upper right**) C-section twist; (**lower left**) Z-section transverse displacement; (**lower right**) Z-section twist.

**Figure 17:**
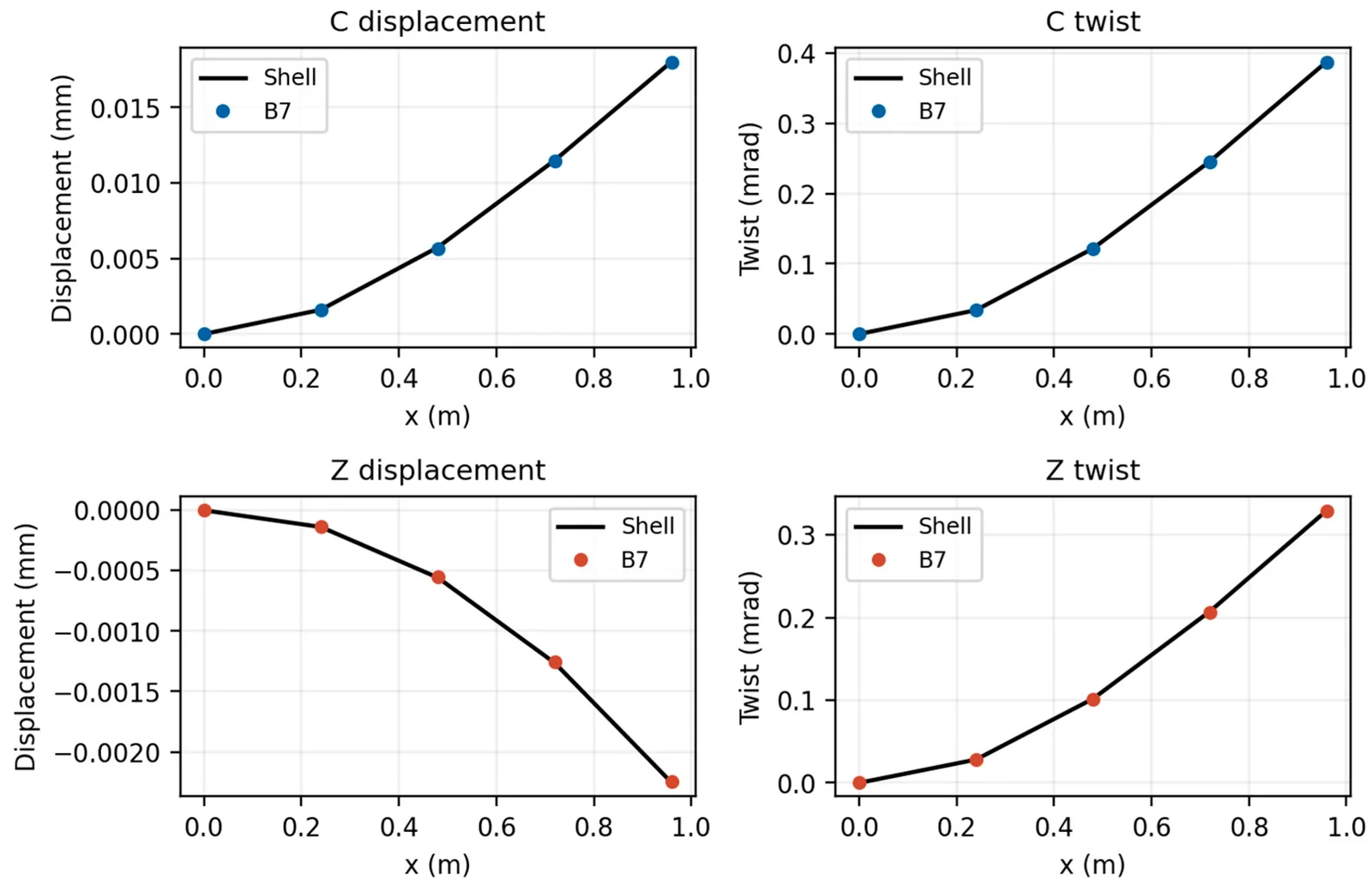
Validation for a non-uniformly perforated member: transverse displacement in the left column and twist in the right column, with C-section results in the upper row and Z-section results in the lower row. Full shell and assembled seven-DOF reduced solutions are compared.

**Figure 18:**
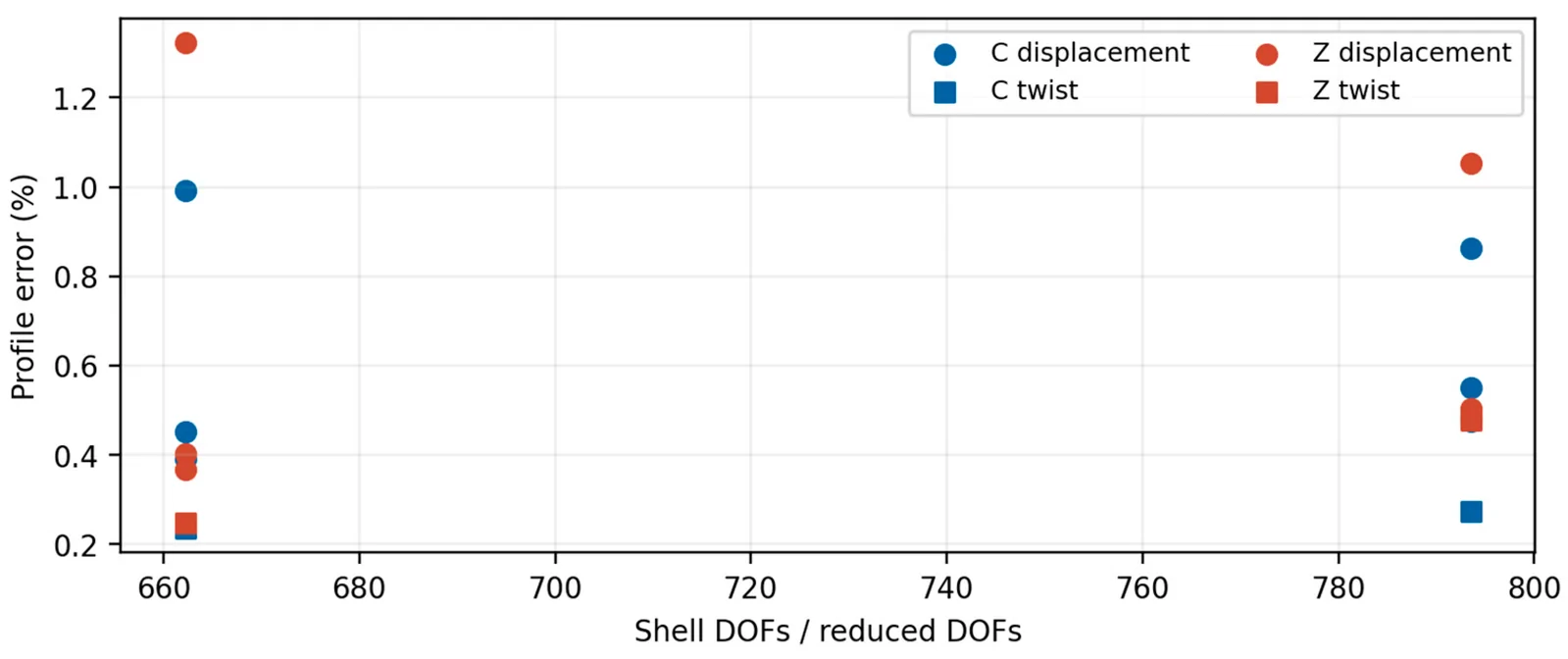
DOF reduction and profile errors for directly condensed B7 models under combined, compression, and shear loading. Each point represents a reported numerical case; the plot does not define a general error bound.

The member comparisons in Figure 16, Figure 17 and Figure 18 use directly condensed matrices to separate structural reduction from regression. Four repeated segments give *L* = 0.96 m; each has *L_e_* = 0.240 m, *α* = 0.60, and *β* = 0.5333. The restrained-warping modal end conditions described above are used, with *M_y_* = 1 N m and *T* = 0.5 N m. The shell model is continuous across the three internal cuts, with no constraints on their deformation or rotations.

For the repeated C-section member, the displacement and twist profile errors are 0.452% and 0.236%; for Z they are 0.367% and 0.248%. The shell/reduced tip displacements are 0.018803/0.018744 mm for C and -0.002372/-0.002363 mm for Z. The corresponding tip twists are 0.397941/0.396952 mrad and 0.371361/0.370505 mrad. Profile errors and separate tip errors are listed distinctly in Table 9.

Each continuous repeated-opening shell model contains 23,178 degrees of freedom, while the B7 assembly contains 35. Counts refer to the global degrees of freedom before application of end restraints. The mesh used for these member comparisons is the same nominal third-level discretization as the segment study.

For the non-uniform test, the four successive opening pairs are (*α*,*β*) = (0.30, 0.30), (0.45, 0.60), (0.60, 0.45), (0.35, 0.75). All segments use the same physical sectorial function and sign convention. Their transverse mesh contains the union of the opening-edge levels so that both the full shell member and its individual segment models use compatible interfaces. The end conditions and loads are unchanged from the repeated-opening case.

The non-uniform C-section displacement and twist profile errors are 0.550% and 0.275%; the Z-section errors are 0.484% and 0.477%. Each continuous shell model contains 27,774 degrees of freedom, compared with 35 for the reduced assembly.

The shell/reduced tip displacements are 0.018043/0.017975 mm for C and −0.002256/−0.002247 mm for Z; tip twists are 0.387938/0.386816 mrad and 0.329785/0.328408 mrad. Additional member tests under *N* = −100 N and *V_z_* = 10 N give maximum displacement-profile errors of 0.504% and 1.324%, respectively, over the two families and both opening patterns. These are initial-stiffness compression and shear-load comparisons, not buckling or resistance calculations. Table 10 summarizes the model sizes and DOF reduction factors.

The direct member models reduce the global DOF count by factors of 662.23 for repeated openings and 793.54 for non-uniform openings. The six-segment torsional comparison gives 706.90 for B7. Across the direct four-segment combined, compression, and shear cases, the maximum displacement/twist profile error is 1.324%. These ratios measure model size; they are not measured run-time acceleration.

Wall-clock measurements use an AMD EPYC 9V74 80-Core Processor, Linux, Python 3.12.14 and SciPy 1.17.0, with one BLAS thread. Medians of three trials for the repeated/non-uniform C/Z members are 4.31–5.51 s for shell meshing, assembly, boundary enforcement and direct solution, versus 0.098–0.106 ms for reduced assembly and solution from available cell matrices. The latter excludes 0.87–4.62 s for generating and condensing the required distinct cells; including this cost gives first-use speedups of 1.14–5.35. Separately, producing the 12 training cells plus the intact reference costs 13.37–13.79 s per family, and POD/GPR fitting costs 0.16–0.18 s. These surrogate-preparation costs are excluded from the direct-member timings. Application-specific shell verification is an additional cost. Post-processing and file I/O are excluded throughout; raw timings are supplied in the numerical supplement.

For the six-segment torque example, B6 increases the DOF reduction factor to 824.71 but its free-warping twist error is 99.870%. The seventh coordinate therefore adds little global dimension while recovering a deformation mechanism that is essential for this interface reduction.

**Figure 19:**
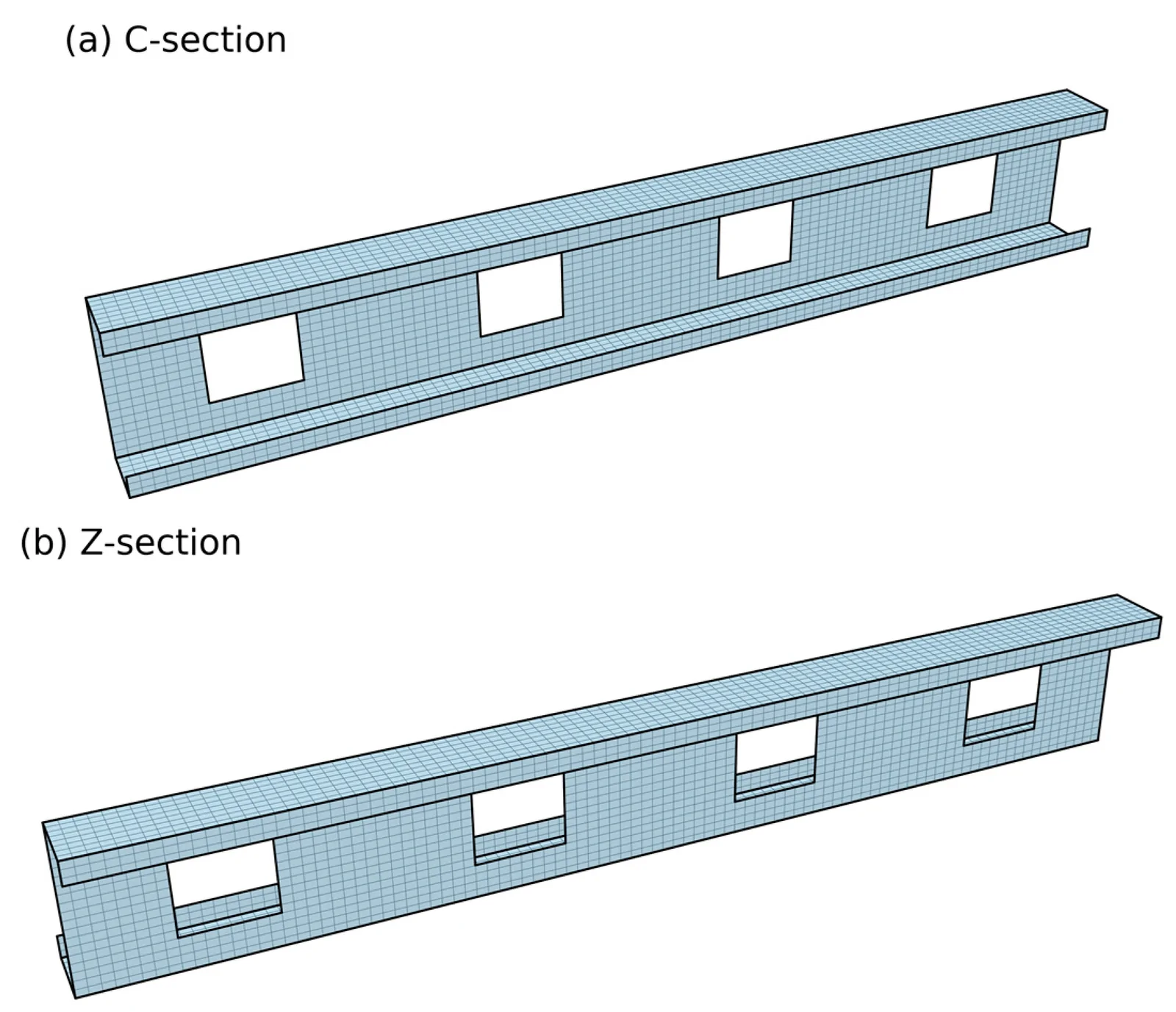
Actual continuous shell meshes for the repeated-opening four-segment reference members in: (**a**) C- and (**b**) Z-section. The two physical end traces use the modal attachments described in Section 3.1. Interior cross-sections share all shell DOFs and are not constrained to the reduced basis. Dimensions are shown in mm.

**Figure 20:**
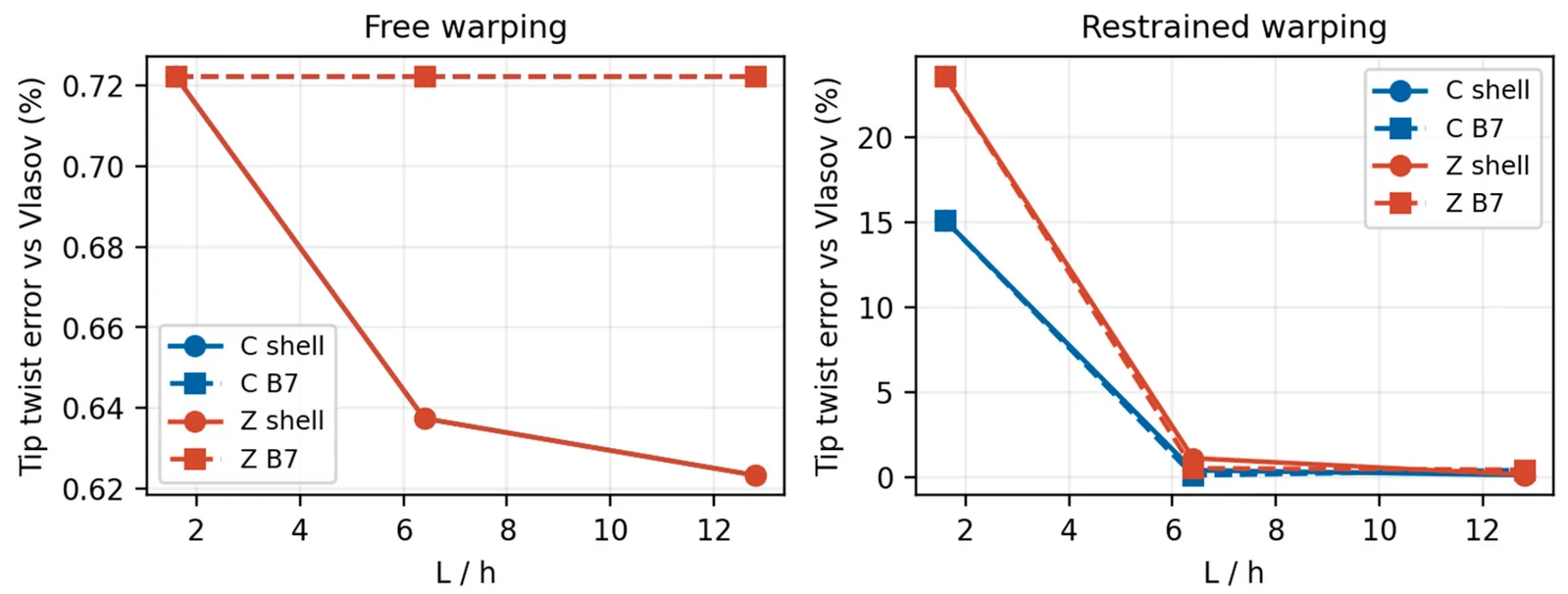
Independent unperforated torsion benchmark: tip-twist errors relative to the closed-form Vlasov solution with free warping in the left panel and restrained warpin in the right panel. Both the continuous shell and B7 predictions are shown, including the short-member discrepancy.

**Figure 21:**
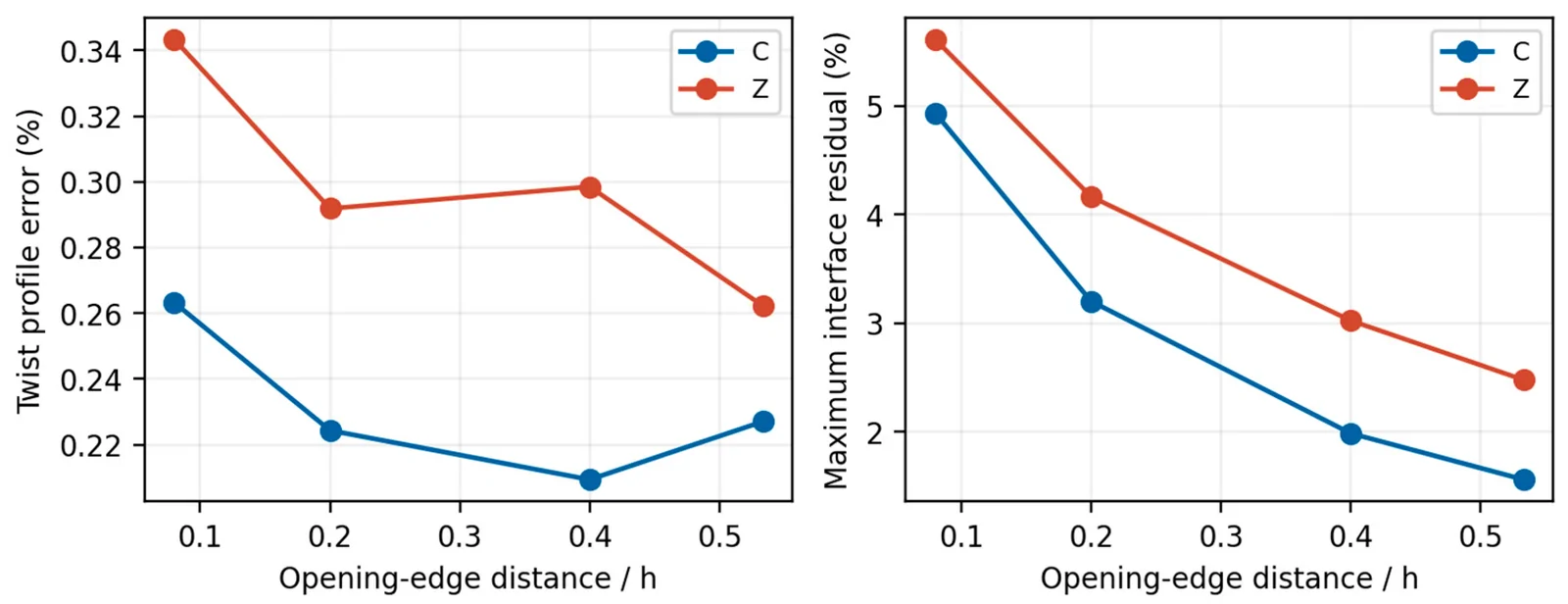
Large openings approaching the interfaces: global twist-profile error in the left panel and maximum weighted interface translation residual in the right panel. All points use h_o_/h = 0.65; the full shell interior remains unconstrained.

**Figure 22:**
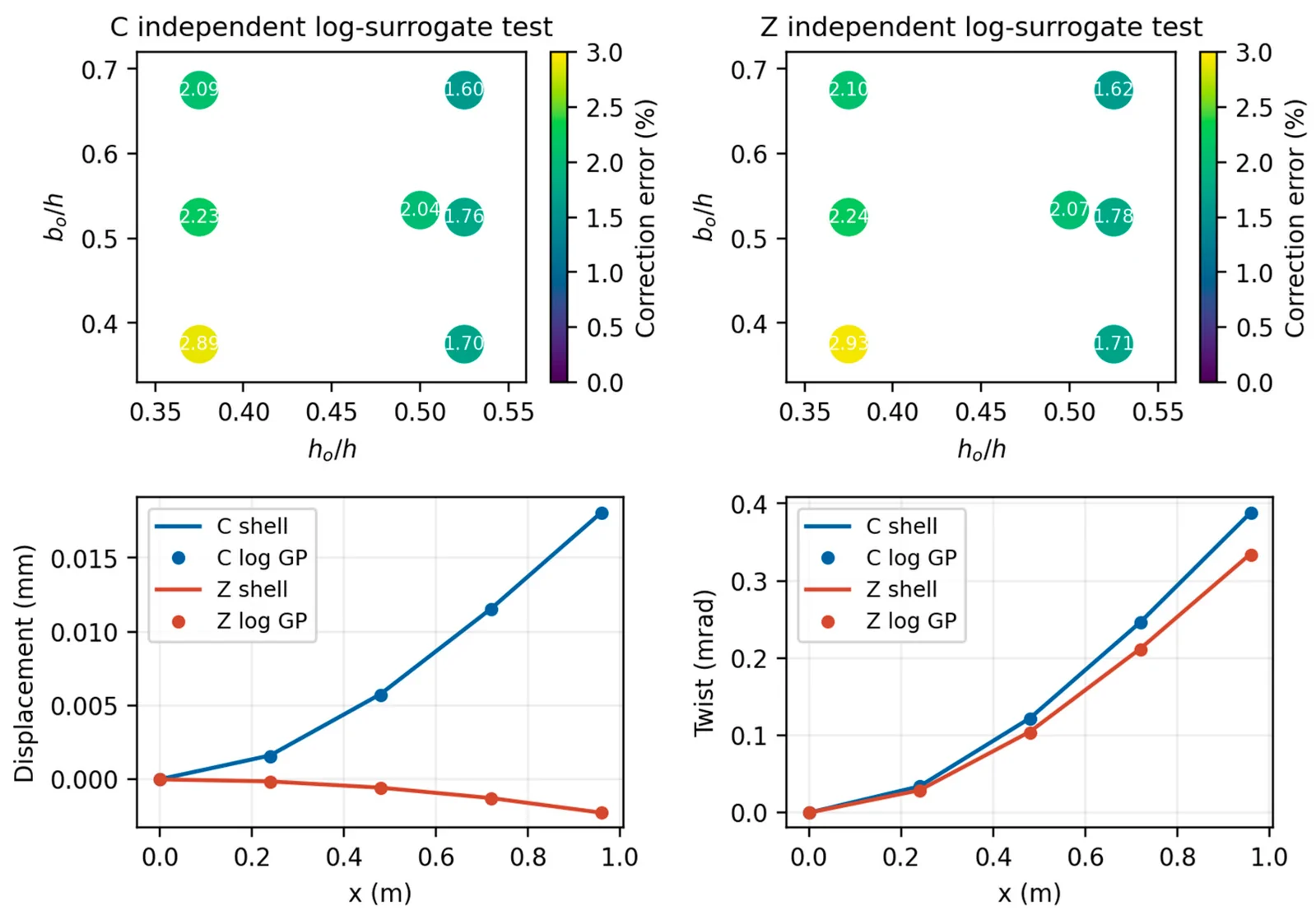
Logarithmic surrogate verification: correction errors at independent off-grid geometries for C-sections (**upper left**) and Z-sections (**upper right**), and full-member displacement (**lower left**) and twist (**lower right**) profiles for four off-grid openings. The shell reference and entirely surrogate-based assembly are compared at the five intact sections.

Figure 19 shows the actual continuous shell meshes for the repeated four-segment examples. The analytical, near-interface, and complete surrogate-to-member checks in Figure 20, Figure 21 and Figure 22 extend verification beyond the direct-condensation comparisons above. Their scripts, inputs, and CSV outputs are supplied with the numerical supplement.

### 3.6. Independent and Additional Verification

The Vlasov comparison in Figure 20 uses the unperforated section constants and boundary conditions derived in Appendix A. For L/h = 6.4 and 12.8, shell tip-twist errors are no larger than 1.104% and B7 errors no larger than 0.723%. The short restrained segment at L/h = 1.6 differs by 15.120% for C and 23.554% for Z. Reporting this case identifies a limitation of the nondistorting beam idealization, including its end-condition interpretation, rather than suggesting equally close agreement at every length.

The near-interface study uses four segments under T = 1 N·m, restrained left-end warping, and a large opening height h_o_/h = 0.65. Opening lengths b_o_/h = 0.5333, 0.80, 1.20, and 1.44 reduce the opening-edge distance from 0.5333h to 0.080h. Figure 21 shows a maximum twist-profile error of 0.343%, but the interface residual reaches 4.935% for C and 5.612% for Z. Small torque-response errors alone do not establish load-independent adequacy of the interface reduction.

For load-independent use, an operational acceptance tolerance of 2% is imposed on the global strain-energy error. With the restrained end attachment of Section 3.1, seven independent tip-resultant analyses provide the 7 × 7 tip-compliance matrices of the continuous shell and B7 models. Every generalized eigenvalue of the B7 matrix relative to the shell matrix must lie between 0.98 and 1.02. This condition bounds the relative energy error for any combination of those tip resultants, including the bimoment, and captures both interface truncation and rotational relaxation. If it fails, the B7 reduction is rejected for this tolerance; a full shell model or an enriched interface with compatible rotations is required. It does not certify local stresses or distortion.

The parameter map comprises 168 C/Z cases with α = 0.30, 0.50, 0.65 and 0.80; d/h = 0.08, 0.20, 0.40, 0.5333, 0.60, 0.65 and 0.70; and L/h = 3.2, 6.4 and 12.8, where d = (L_e_ − b_o_)/2 and L_e_/h = 1.6. Across both families and the sampled α ≤ 0.65, the smallest sampled clearances satisfying the 2% criterion throughout this height range are d/h = 0.70, 0.65 and 0.60 at the three respective slenderness ratios. At α = 0.80, d/h = 0.70 still fails for L/h = 3.2 and 6.4 (maximum errors 2.63% and 2.14%), but passes at L/h = 12.8. Conversely, α = 0.65, d/h = 0.08 and L/h = 6.4 gives an energy error up to 66.43%, despite a torque-only twist error below 0.35%. These sampled geometric bounds guide screening; the eigenvalue criterion governs acceptance between sampled points. The complete map is provided in the numerical supplement.

The independent logarithmic-surrogate tests use six cell-center geometries, h_o_/h ∈ {0.375, 0.525} and b_o_/h ∈ {0.375, 0.525, 0.675}, plus (0.50, 0.5333), for each section family. None is included in the 12-point training grid. The end-to-end member uses the four pairs (0.375, 0.375), (0.525, 0.525), (0.375, 0.675), and (0.525, 0.375), with the same restrained attachment and loads Mᵧ = 1 N·m and T = 0.5 N·m as the direct-member test. All four element matrices are predicted without fallback. Displacement/twist profile errors against the continuous shell are 0.425%/0.211% for C and 0.303%/0.468% for Z. Against the directly condensed reduced member, the corresponding errors are 0.099%/0.062% and 0.130%/0.085%. This separates regression effects from the remaining shell-to-member approximation.

## 4. Discussion

A web opening modifies the generalized stiffness non-uniformly. For the representative opening, the complete dimensionless correction is 28.452% of the intact matrix for C and 28.064% for Z. A single scalar multiplier therefore loses the distribution of these changes among deformation states and coupling terms. The explicit energy-state definitions are essential: a normalized diagonal or prescribed-state energy is not automatically an uncoupled physical section property.

Among the individual deformation mechanisms, transverse shear was consistently the most sensitive to the presence of an opening. Increasing the opening height to *h_o_* / *h* = 0.65 reduced the corresponding normalized stiffness measure to approximately 0.24, whereas the bending-related stiffness remained close to 0.96. A similar tendency was observed when the longitudinal dimension of the opening was increased. At *b_o_*/*h* = 0.80, the shear-related stiffness decreased to approximately one third of the unperforated value, while the bending-related measure remained above 0.98. This distinction is mechanically reasonable because the opening directly interrupts the web region responsible for transmitting shear, whereas global bending stiffness remains dominated by the flange system and by material located farther from the neutral axis.

The C/Z differences arise from their different contour geometry, coupling, and distribution of axial warping and torsional energy. A centered opening can remove a different fraction of the energy of the same generalized state in the two families. The observed torsion ratios of 0.770 and 0.955 at the largest height are specific to the chosen constrained torsion state. The analytical free-warping benchmark instead permits the sectorial coordinate to relax. These two tests answer different questions and should not be interpreted as competing estimates of a single scalar torsion constant.

Retaining additional shell variables cannot reduce the information in the exact Schur complement. The over-stiff response appears when those rotations are subsequently tied to a restrictive rigid-section map. Energy-minimizing rotational lifting reproduces the translation-only result to numerical precision. However, eliminating rotations separately before member assembly relaxes their compatibility across segment cuts; the continuous-shell comparison measures this effect together with the interface restriction. The present results support the chosen reduced model for the tested cases, not universal optimality of translation-only retention.

Sectorial warping is essential for the adopted interface model: B6 torsional errors approach 99%, while B7 gives 0.102–0.253% in the six-segment torque tests. The near-interface study adds an important qualification. Even when the global twist error remains below 0.35%, the omitted interface translation field can reach 5.61%. Agreement in a global response therefore does not justify claims about local distortion, corner stresses, or stability.

The formulation is complementary to extended GBT [22,23,24,25,26,27,28,29,30]. GBT explicitly organizes the deformation in sectional modes and can support analyses beyond the present linear stiffness setting. Here, the perforation interior is represented by a shell mesh and transferred through its condensed response, so opening-specific analytical enrichment functions are not required inside the segment. This can simplify repeated preprocessing of new opening geometries when a suitable shell model is already available. It does not remove the need to choose and verify an interface basis: seven fixed modes remain an approximation. The advantage is therefore a different route to constructing coupled reduced operators, rather than demonstrated superiority to GBT in accuracy or computational time.

The original correction matrices remain highly compressible in the Frobenius norm: two modes per family or three combined modes exceed 99.9% of their snapshot energy. This observation concerns representation of the dominant entries. It does not imply that the same truncation preserves the much smaller torsional stiffness eigenvalues. The failed linear-surrogate checks demonstrate that matrix compression and mechanically reliable prediction must be verified separately.

The logarithmic construction addresses that distinction by scaling to the intact energy and working only on the eight-dimensional deformation space. It preserves positivity and the six rigid modes by construction, while still using three coefficients per family in this database. Its independent off-grid correction errors reach 2.93%, compared with 0.91% in nested leave-one-out testing; the independent test is therefore the more conservative indication of interpolation accuracy. No extrapolation claim is made.

Small Frobenius error and positive definiteness alone are insufficient deployment criteria. The screened linear surrogate remains inaccurate in the member test, whereas the logarithmic model gives displacement/twist profile errors below 0.47% without invoking a fallback in that test. The latter is an end-to-end result for a specified member and load case, rather than evidence that every possible load response is equally accurate. The additional worst-state energy metric helps expose errors that an overall matrix norm can hide.

Direct assembly gives 35 generalized DOFs instead of 23,178–27,774 shell DOFs for the four-segment members. Combined-load profile errors remain below 0.55%, and the added compression and shear cases raise the maximum displacement-profile error to 1.324%. The non-uniform and off-grid surrogate examples require no member-level calibration. These findings support efficient initial-stiffness screening within the investigated geometry and boundary-condition domain.

For non-uniform assembly, one implementation detail deserves attention. The sectorial warping function is defined only up to a multiplicative sign. Independently generated reduced segments may therefore contain equivalent warping modes with opposite orientation. A common sign convention must be enforced before adjacent matrices are assembled. Once this orientation was made consistent, the non-uniform C- and Z-section models reproduced the shell solutions with the expected accuracy. This requirement is straightforward computationally but important for any automated library of reduced perforated elements.

The three intended engineering uses differ in what has been demonstrated. For preliminary stiffness assessment, the member comparisons show that the directly condensed operators reproduce the tested shell displacement and twist profiles with few generalized unknowns. A purlin, rack component, or photovoltaic support with comparable openings could use this level of analysis to assess elastic deflection and twist, while connections, loads, restraints, and resistance still require separate modeling. For geometry screening and sensitivity studies, the two-parameter correction database provides a compact interpolation model, with the validation limitations stated above. For optimization or uncertainty propagation, repeated reuse of a verified element library could reduce the cost of structural solves, but no complete optimization or probabilistic study is reported. The timings in Section 3.5 distinguish first-use and reusable-matrix costs. Offline shell meshing, condensation, database construction, and regression fitting must be included when assessing the overall computational benefit.

The formulation applies only to small-displacement, pre-buckling elastic stiffness. It does not predict yielding, local or distortional buckling, global instability, post-buckling response, connection failure, or ultimate resistance. Loads are kept small in the revised examples and characterize linear compliance; no strength or buckling margin is established. Mesh convergence is component-dependent: the third-mesh shear-state energy still differs by 3.288% from the finest reference. Global response agreement is not a convergence guarantee for local stresses.

Evidence is limited to the specified C/Z dimensions, centered rectangular openings, and the documented modal end attachments. The surrogate is fitted to 12 geometries per family and tested at seven additional geometries per family; its independent data set is still small. Other opening families, eccentricity, edge stiffening, connections, material properties, and section ratios require additional shell evaluations and renewed basis assessment. The clearance map in Section 3.6 provides screening bounds, while the 2% energy criterion determines operational acceptance. The short restrained-warping analytical benchmark also exposes differences between shell behavior and a nondistorting Vlasov idealization. Experimental stiffness validation remains outside this numerical study.

Existing experiments provide qualitative context but require a different comparison protocol. Uzzaman et al. [8] studied web crippling under concentrated bearing loads, including flange fastening, whereas Pham et al. [16] investigated shear strength and its dependence on web-hole geometry. These studies support the importance of opening geometry and loading conditions, but ultimate loads and failure modes cannot validate an initial torsion/warping stiffness. A quantitative stiffness comparison would require the measured geometry, bearing/contact and restraint conditions, and the initial load–displacement slopes to be reproduced. That mapping is not established here; the present modal end attachments do not represent those test rigs.

## 5. Conclusions

A reduced stiffness formulation for perforated thin-walled C- and Z-sections was developed by combining shell finite element analysis, static condensation, enriched beam-type interface kinematics, proper orthogonal decomposition, and surrogate modeling. The study leads to several main conclusions.

The translation-only B7 interface preserves the dominant warping compliance of the tested members. In the six-segment torque case, B7 twist-profile errors are 0.102% with free warping and 0.253% with restrained warping; the B6 errors approach 99%. Same-mesh condensation and reaction routes agree to about 10^−13^, and energy-optimal rotational lifting confirms that the rotational projection, rather than retention itself, causes artificial stiffening.

Opening effects are strongly non-uniform: the representative correction is approximately 28% of the intact matrix. At *α* = 0.65, the shear-related energy ratio is about 0.244 while the selected bending ratio remains about 0.957. Torsion, warping, and coupling ratios require explicitly stated displacement states and cannot be treated as interchangeable scalar section properties.

Three linear POD modes capture more than 99.9% of the combined correction-matrix snapshot energy, but their small Frobenius errors do not ensure admissible or accurate member stiffness. The energy-scaled logarithmic surrogate retains three coefficients per family, preserves the six rigid modes and positive deformational stiffness, and gives a maximum independent off-grid correction error of 2.932% and a maximum worst-state energy error of 3.646%.

Independent unperforated Vlasov benchmarks give shell tip-twist errors no larger than 1.104% for the tested *L*/*h* = 6.4 and 12.8 members. The short *L*/*h* = 1.6 restrained case differs by 15.120% for C and 23.554% for Z, demonstrating a limit of the nondistorting beam comparison. Near-interface openings give global twist errors below 0.35% but interface residuals up to 5.61%; local distortion accuracy is therefore not inferred from global agreement.

Direct four-segment models reduce the DOF count by factors of 662–794, with maximum displacement/twist profile error of 1.324% across the tested combined, compression, and shear cases. A separate off-grid member assembled entirely from logarithmic-surrogate matrices gives profile errors below 0.47%. These profile errors refer to the specified load cases; load-independent acceptance requires the 2% energy check in Section 3.6. DOF ratios and measured first-use speedups are reported separately.

The resulting framework provides a reproducible reduced stiffness model for preliminary elastic assessment, opening-geometry screening, and repeated analyses within a verified parameter domain. It complements strength and stability methods. The complete numerical pipeline, including failed linear-surrogate diagnostics and the corrected logarithmic predictor, is supplied so that the quantitative claims can be checked. Physical validation and extension beyond the tested domain remain necessary for broader application.

## Figures and Tables

**Table 1:** Geometric and material quantities used in the reduced-stiffness formulation.

Symbol	Definition	Dimensionless Form/Comment
*h*	clear web height	characteristic length *L*_∗_ = *h*
*b_f_*	flange width	*b_f_*/*h*
*c_l_*	lip length, when present	*c_l_*/*h*
*t*	wall thickness	*t*/*h*
*L_e_*	reduced-element length	*λ* = *L_e_*/*h*
*h_o_*	opening height	*α* = *h_o_*/*h*
*b_o_*	opening length in the longitudinal direction	*β* = *b_o_*/*h*
*e_o_*	vertical eccentricity of the opening center	*ε* = *e_o_*/*h*
*r_o_*	corner radius, when applicable	*ρ* = *r_o_*/min(*h_o_*,*b_o_*)
*E*	Young’s modulus	linear elastic material parameters
*ν*	Poisson’s ratio	

**Table 2:** Reduced interface bases considered for assessment of the warping enrichment.

Model	Generalized Coordinates at Each End	Element Matrix Size	Purpose
B6	*u*, *v*, *w*, *θ_x_*, *θ_y_*, *θ_z_*	12 × 12	classical rigid-section reference
B7	*u*, *v*, *w*, *θ_x_*, *θ_y_*, *θ_z_*, *γ_ω_*	14 × 14	proposed warping-enriched model

**Table 3:** Validation hierarchy and representative load cases.

Level	Compared Models	Representative Actions	Main Quantities Assessed
1	B6/B7 and continuous shell; analytical Vlasov limit	axial force, major/minor bending, shear, torsion, combined bending-torsion	energy, twist, interface residual; free/restrained warping
2	Linear baseline and logarithmic POD–GPR versus direct matrices	Nested leave-one-out and 14 independent off-grid geometries	matrix error, worst-state energy error, full-element admissibility
3	assembled reduced member, full shell member	combined bending–torsion, axial compression, transverse force	profiles, energy, direct and surrogate-based assembly

**Table 4:** Mesh-convergence study for the reference and representative perforated segments.

Model	Mesh	Δ*x*/*h*	Δ*s*/*h*	Nodes	Shell Elements	Shell DOFs	Relative Error of *K_e_* [%]
Solid C	1	0.1333	0.1000	299	264	1794	0.288
Solid C	2	0.1000	0.0667	578	528	3468	0.143
Solid C	3	0.0667	0.0500	1075	1008	6450	0.047
Solid C	4	0.0500	0.0333	2145	2048	12,870	Reference
Perforated C	1	0.1333	0.1000	284	240	1704	1.418
Perforated C	2	0.1000	0.0667	606	540	3636	0.559
Perforated C	3	0.0667	0.0500	998	912	5988	0.325
Perforated C	4	0.0500	0.0333	2088	1960	12,528	Reference

**Table 5:** Influence of interface representation and warping enrichment on reduced-element accuracy.

Interface Shell DOFs per Node	Generalized DOFs per Reduced Node	Axial Error [%]	Major-Axis Bending Error [%]	Minor-Axis Bending Error [%]	Torsional Error [%]	Bending–Torsion Error [%]	Mean Energy Error [%]
3	6	0.423	0.621	0.276	99.870	99.819	40.202
3	7	0.423	0.507	0.276	0.102	0.102	0.282
6	6	0.443	0.648	0.303	99.871	99.819	40.217
6	7	0.443	0.543	0.303	82.142	82.100	33.106

**Table 6:** Magnitude of the opening-induced stiffness correction for the representative centered rectangular opening.

Section	Opening Shape	*h_o_*/*h*	*b_o_*/*h*	*e_o_*/*h*	Relative Correction [%]
C-section	Rectangular	0.600	0.533	0	28.452
Z-section	Rectangular	0.600	0.533	0	28.064

**Table 7:** POD compression of the stiffness-correction database.

Section/Data Set	Snapshots	Modes for 95% Energy	Modes for 99% Energy	Modes for 99.9% Energy	Adopted *R*	Mean Reconstruction Error [%]	Maximum Reconstruction Error [%]
C	12	1	1	2	2	0.888	2.069
Z	12	1	1	2	2	1.300	3.092
C+Z	24	1	2	3	3	1.468	3.004

**Table 8:** Surrogate accuracy for the POD coefficients and stiffness corrections, including verification of the logarithmic formulation.

Accuracy metrics of the surrogate model for the POD coefficients and the reconstructed reduced stiffness correction.										
Quantity		RMSE	NRMSE [%]			*R* ^2^	Mean matrix error [%]		Maximum matrix error [%]	
a1		0.000645	0.220			0.999947	–		–	
a2		0.000169	0.156			0.999978	–		–	
a3		0.000738	0.929			0.998806	–		–	
Linear correction		–	–			–	1.517		3.166	
Mean and maximum reconstruction errors of the surrogate-predicted reduced stiffness correction for the C- and Z-sections.										
Section		Mean matrix error [%]					Maximum matrix error [%]			
C		1.397					2.371			
Z		1.637					3.166			
Verification of the logarithmic surrogate; correction and worst-state energy errors are percentages.										
Section	POD rank	Nested mean		Nested max	Test mean		Test max	Energy max		Invalid test
C	3	0.224		0.911	2.045		2.894	3.645		0/7
Z	3	0.239		0.886	2.066		2.932	3.643		0/7

**Table 9:** Validation of the assembled reduced model against the complete shell model.

Quantity	C-Section	Z-Section
Full shell DOFs	23178	23178
Reduced DOFs	35	35
Tip displacement, shell (mm)	0.018803	−0.002372
Tip displacement, reduced (mm)	0.018744	−0.002363
Tip displacement error (%)	0.316	0.381
Tip twist, shell (mrad)	0.397941	0.371361
Tip twist, reduced (mrad)	0.396952	0.370505
Tip twist error (%)	0.249	0.231
Profile error w (%)	0.452	0.367
Profile error twist (%)	0.236	0.248

**Table 10:** Computational efficiency of the proposed reduced framework.

Validation Case	Reduced Formulation	Full Shell DOFs	Reduced DOFs	DOF Reduction Factor	Mean Profile Error [%]	Maximum Profile Error [%]
Repeated, C	B7	23178	35	662.23	0.344	0.452
Repeated, Z	B7	23178	35	662.23	0.308	0.367
Nonuniform, C	B7	27774	35	793.54	0.412	0.550
Nonuniform, Z	B7	27774	35	793.54	0.480	0.484
Free warping	B7	34638	49	706.90	0.102	0.102
Restrained warping	B7	34638	49	706.90	0.253	0.253
Free warping	B6	34638	42	824.71	99.870	99.870
Restrained warping	B6	34638	42	824.71	98.596	98.596

## Data Availability

The original contributions presented in this study are included in the article. Further inquiries can be directed to the corresponding author.

## Appendix A. Classical Torsion Reference for an Unperforated Member

An independent analytical reference is obtained for a straight, prismatic, unperforated member under linear Vlasov torsion. The contour is the same as in the shell geometry, without corner radii or thickness-overlap corrections. Exact line integration gives *A*_0_ = 6.20 × 10^−4^ m^2^ and *J* = *t*^3^(*h* + 2*b_f_* + 2*c_l_*)/3 = 8.267 × 10^−10^ m^4^ for both families. The orthogonalized warping constants are *I_ω_* = 1.726410 × 10^−9^ m^6^ for C and 2.260877 × 10^−9^ m^6^ for Z. The classical torsion/warping energy [41] and the following closed-form solutions are independent of the shell condensation algorithm.

(A1) $$U=\frac{1}{2}\int_{0}^{L}\left[\mathrm{GJ}{\left(\theta^{\prime }\right)}^{2}+EI_{\omega }{\left(\theta \prime \prime \right)}^{2}\right]\mathrm{dx}.$$

Stationarity under an end torque *T* and zero distributed torque gives *EI_ω_θ*^(4)^ − *GJθ*^(2)^ = 0. The end torque is *GJθ*^(1)^ − *EI_ω_θ*^(3)^, and zero warping traction corresponds to *EI_ω_θ*^(2)^ = 0. With twist fixed at *x* = 0 and warping free at both ends, the solution is

(A2) $$\theta \left(x\right)=\frac{\mathrm{Tx}}{\mathrm{GJ}},\;\;\theta \left(L\right)=\frac{\mathrm{TL}}{\mathrm{GJ}}.$$

With twist and warping restrained at the left end, *θ*(0) = *θ*′(0) = 0, and zero warping traction at the torque-loaded right end, define $\kappa =\sqrt{\mathrm{GJ}/\left(EI_{\omega }\right)}$. Integration gives

(A3) $$\theta \left(x\right)=\frac{T}{\mathrm{GJ}}\left[x-\frac{\sinh\left(\mathrm{\kappa x}\right)}{\kappa }+\frac{\tanh\left(\mathrm{\kappa L}\right)}{\kappa }(\cosh\left(\mathrm{\kappa x}\right)-1)\right],$$

(A4) $$\theta \left(L\right)=\frac{T}{\mathrm{GJ}}\left[L-\frac{\tanh\left(\mathrm{\kappa L}\right)}{\kappa }\right].$$

Equations (A2)–(A4) are evaluated at *T* = 1 N m and *L* = 0.24, 0.96, and 1.92 m and compared quantitatively with both continuous shell and B7 models in Figure 20 and Table A1. For the longer members, shell errors are at most 1.104% and reduced-model errors at most 0.723%. The short restrained segment has larger errors, 15.120% for C and 23.554% for Z; these are retained in the comparison. Vlasov theory assumes a nondistorting section, whereas the shell permits local deformation and uses the specified modal attachment with free shell rotations. Agreement for longer unperforated members therefore verifies a classical limit; it is not an analytical solution for perforated members or a substitute for experiment.

**Table A1:** Independent torsion benchmark under T = 1 N·m. Twist is in mrad; errors are relative to Vlasov theory.

Section	L (m)	Warping	Theory	Shell	B7	Shell Error (%)	B7 Error (%)
C	0.24	free	3.59447	3.62043	3.62043	0.722	0.722
C	0.24	restrained	0.01266	0.01457	0.01457	15.120	15.120
C	0.96	free	14.37788	14.46952	14.48172	0.637	0.722
C	0.96	restrained	0.76177	0.76478	0.76250	0.395	0.096
C	1.92	free	28.75576	28.93499	28.96344	0.623	0.722
C	1.92	restrained	5.12120	5.11554	5.10061	0.111	0.402
Z	0.24	free	3.59447	3.62043	3.62043	0.722	0.722
Z	0.24	restrained	0.00967	0.01195	0.01195	23.554	23.554
Z	0.96	free	14.37788	14.46952	14.48172	0.637	0.722
Z	0.96	restrained	0.59055	0.59707	0.59357	1.104	0.511
Z	1.92	free	28.75576	28.93499	28.96344	0.623	0.722
Z	1.92	restrained	4.11743	4.12273	4.10002	0.129	0.423
